# Long-Term Mississippi River Trends Expose Shifts in the River Load Response to Watershed Nutrient Balances Between 1975 and 2017

**DOI:** 10.1029/2021wr030318

**Published:** 2021-11-02

**Authors:** Sarah Stackpoole, Robert Sabo, James Falcone, Lori Sprague

**Affiliations:** 1 U.S. Geological Survey, Denver, CO, USA; 2 U.S. Environmental Protection Agency, Washington, DC, USA; 3 U.S. Geological Survey, Reston, VA, USA

## Abstract

Excess nutrients transported by the Mississippi River (MR) contribute to hypoxia in the Gulf of Mexico. Nutrient balances are key drivers to river nutrient loads and represent inputs (fertilizer, manure, deposition, wastewater, N-fixation, and weathering) minus outputs (nutrient uptake and removal in harvest, and N emissions). Here, we quantified annual changes in nitrogen (N) and phosphorus (P) river loads and nutrient balances at the MR Outlet and documented that the river load response to watershed nutrient balances shifted between 1975 and 2017. Annual nutrient balances and river loads were positively correlated between 1975 and 1985, but after, a disconnect between both the N and P balances and river loads emerged, and the subsequent river load patterns were different for N versus P. We evaluated the relative impacts of legacy nutrients and other latent factors, for which data were not available, on river nutrient load trends. Our analysis showed that in the case of N, latent factors were potentially just as important in explaining changes in river nutrient loads over time as N balances, and in the case of P, they were even more important. We hypothesized that these factors included implementation of best management practices, changes in watershed buffering capacity, the effects of tile drainage, or increased precipitation. Our analytical approach shows promise for the investigation of drivers of water quality trends that are not well-represented in typical national scale geospatial datasets.

## 1. Introduction

The hypoxic zone in the northern Gulf of Mexico (GoM) is greatly influenced by the high nutrient loads from the Mississippi River (MR) (Boesch et al., 2009; Lohrenz et al., 2008; Turner & Rabalais, 2003). N and P delivery to the GoM is shaped by nutrient use and management within the MR Basin, which spans approximately 1.9 million km^2^ (Alexander et al., 2008; Goolsby et al., 1999; McIsaac et al., 2002). In the basin, management to minimize the negative impacts of excess nutrients on surface waters has taken on many forms, including improvement to the timing and rate of fertilizer and manure applications for plant uptake, implementation of best management practices (BMPs), tillage management to reduce erosion, enhancement of nutrient application products such as urease and nitrification inhibitors, and technology upgrades at wastewater treatment facilities (United States Department of Agriculture, 2012, 2013). The aggregate effects of some nutrient management are captured in watershed nutrient balances. Balances equal the difference between inputs (fertilizer, manure, deposition [for N], wastewater effluent, fixation [for N], and weathering [for P]) and outputs (nutrient uptake and removal in harvest, soil emissions [for N], and river surface emissions [for N]) and indicate if there are N and P surpluses (inputs exceed outputs) or deficiencies (outputs exceed inputs). Management that includes adjustments to the rate and timing of fertilizer or manure applications may result in lower annual nutrient balances.

In theory, a reduction in nutrient balances should produce a reduction in river nutrient loads. However, reductions in nutrient balances through time, even into deficiencies, have been linked to stable or even increasing river loads (Haygarth et al., 2014; Powers et al., 2016). This may be due to legacy nutrients, which are defined as historical N and P from fertilizer and manure applications, wastewater discharges, or atmospheric deposition that has accumulated in a watershed over time. Therefore, although the expected consequence of reductions in nutrient balances is improved water quality, or a reduction in river loads, remobilization of legacy (historical) nutrients from the land surface, groundwater, the river floodplain, or channel, can contribute to increased loads and obscure any improvements in the contemporary (current year) water quality signal.

Other factors may also cause a disconnect between changes in nutrient balances and river loads. Recent trend studies have shown that even in watersheds where legacy nutrients were likely sources and nutrient balances increased, no consistent degradation in river water quality occurred (Stackpoole et al., 2019). It was hypothesized that this pattern may be due to the implementation of BMPs, or the effect of watershed buffering capacity. Implementation of BMPs can reduce the transport of surplus nutrients from fields to streams, even when nutrient balances are increasing. Watershed buffering capacity characterizes the absorption of excess nutrients in soils, groundwater, riparian areas, or streambeds without causing any noticeable difference in river nutrient loads (Carpenter et al., 1998; Doody et al., 2016; Fraterrigo & Downing, 2008; Kleinman, 2017). An abrupt upward shift in river loads despite relatively static or even decreasing nutrient balances is a proxy that has been used to identify watersheds where a buffering capacity has been exceeded (Goyette et al., 2018). Currently, comprehensive national-scale data on BMP implementation or direct estimates of differences in watershed buffering capacity are not available, making it difficult to link the effects of these factors to changes in river loads.

Here, we estimate annual river sediment loads, river N and P loads, and N and P balances near the outlet of the MR, hereafter referred to as the MRO. Balances were estimated for the period from 1950 to 2017, in part pre-dating the development of large-scale hypoxia, which did not form consistently until the mid- to late-1970s (Scavia & Donnelly, 2007). Due to more limited data availability, river load estimates spanned a shorter period from 1975 to 2017. Our first objective was to determine changes in river loads and nutrient balances over time. Our second was to explore possible causes of these changes. Determining the causes of nutrient trends at the MRO is challenging because of the number of potential causal factors in this large basin and limited data availability. Previous work used time lags of nutrient balances and river discharge to explain 95% of the variability in river nitrate loads from 1960 to 1998 (McIsaac et al., 2002). This study serves as a continuation and expansion of that work, as we developed an updated regression model that explained the variability in N and P and explored additional variables, including both legacy and current nutrient sources and a latent variable that represented other watershed processes that are not captured in available data sets, but may have been influencing nutrient load trends. We augmented our analysis, which was initially informed by the regression model results, and used counterfactual scenarios to compare observed changes in river loads to potential changes in river load that might have occurred in the absence of changes nutrient balances, changes in legacy sources, or changes in the latent variable. With this analysis, we were able to evaluate the relative impact of time lags and latent variables on nutrient loads, while also building reasonable hypotheses about what the latent variables represent.

## 2. Methods

### 2.1. River Nutrient Load and Concentrations Trends

Annual river loads and trends were estimated for the MRO, which is located near the mouth of the MR but upstream of the Old River Control Structure, which diverts approximately one-third of the flow from the MR into the Atchafalaya River (Murphy et al., 2013; Sprague et al., 2011) (Figure S1 in Supporting Information S1). Loads and trends were estimated from 1975 to 2017 and for three 10-year and one 12-year increments (1975–1985, 1985–1995, 1995–2005, 2005–2017) for five water quality constituents: TP (total phosphorus), OP (orthophosphate), TN (total nitrogen), NO23 (nitrite plus nitrate), and NH3 (ammonia) (Table S1 in Supporting Information S1). Suspended sediment (SS) loads and trends were estimated from 1979 to 2017.

The weighted regression on time, discharge, and season (WRTDS) model was used to estimate annual river loads (kg year^−1^), concentrations (mg L^−1^), and the change in river loads (%) between 1975 or 1979 (for SS loads only) and 2017 (Hirsch et al., 2010). We used the flow normalized (FN) loads and concentrations, as well as the “actual” (i.e., true condition) load estimated by WRTDS. Flow normalization removes the effects of random changes in streamflow to better elucidate the impacts of changes in point, groundwater, and nonpoint sources on both loads and concentration. “Actual” loads were estimated using an extension of WRTDS (WRTDS_K), which uses Kalman filtering to account for serial autocorrelation in daily load estimates; this removes the constraint of homoscedastic residuals to better represent the temporally varying seasonal and streamflow-related water-quality patterns (Zhang & Hirsch, 2019). These “actual” loads (hereinafter referred to as Kalman filter loads) are not flow-normalized. The FN loads were also used to estimate trends in nutrient loads and served as the dependent variable in the regression model and the counterfactual analysis. The FN concentrations were used to estimate the molar ratios of TN and TP and evaluate changes in stoichiometry. The Kalman filter estimates were used to estimate nutrient retention in the watershed.

A bootstrap method supplied a likelihood statistic for the estimated FN load trend (Hirsch et al., 2015), which is equivalent to the two-sided *p*-value, and (a) indicated whether the null hypothesis, that there is no trend over the period of record, should be rejected, and (b) provided a measure of the strength for the estimated trend. Upward or downward trends were considered “likely” if the likelihood was >0.85, “somewhat likely” if the likelihood was from 0.85 to 0.70, and “as likely as not” to be upward or downward if the likelihood was <0.70.

Model performance evaluations, including a visual assessment of the model fit and the detection of bias in residuals were completed, and no models showed problems in either. The WRTDS models to produce FN load, Kalman filter load, and FN concentration estimates were run at a daily time step, and the loads and concentrations were aggregated to annual means. The FN and Kalman filter load estimates were converted to common units (kg km^−2^) by dividing the river load by the upstream drainage area. The molecular weights of N (*n* = 14) and P (*N* = 32) were used to convert the TN and TP concentrations in mg L^−1^ of N and P, respectively, to molar ratios. More details about river load estimation and trend analysis methods are available in Section S2 in Supporting Information S1, Methods—River Loads. The estimated changes in the concentration-discharge relationship over time were evaluated using WRTDS contour plots, which showed time (*x*-axis), discharge (*y*-axis), and concentrations (*z*-axis, color gradient) for TN, NO23, TP, and OP.

### 2.2. Nutrient Balances

Annual N and P balances from 1950 to 2017 were estimated for the MR Basin (MRB), upstream of the MRO (drainage area of 2,887,854 km^2^) (Figure S2 in Supporting Information S1) using Equations 1 and 2.

The N and P balances were calculated as:

(1) $$N\;\text{balance}\left({\text{kg km}}^{-2}\right)=\left(N_{\text{fertilizer}}+N_{\text{manure}}+N_{\text{wastewater}}+N_{\text{fixation}}+N_{\text{atmospheric deposition}}\right)-\;\left(N_{\text{cropuptake}}+N_{\text{emissions}}\right)$$

(2) $$P\;\text{balance}\left({\text{kg km}}^{-2}\right)=\left(P_{\text{fertilizer}}+P_{\text{manure}}+P_{\text{wastewater}}+P_{\text{weathered rock}}\right)-\left(P_{\text{cropuptake}}\right)$$

The balances were calculated as *inputs* (fertilizer, manure, wastewater treatment facility effluent, N_2_O fixation [for N], atmospheric deposition [for N], and weathering [for P]) minus *outputs* (crop uptake and removal in harvest and gaseous emissions from soils and rivers to the atmosphere [for N]). The fertilizer and manure data were based on fertilizer sales in both “farm” and “nonfarm” settings (Brakebill & Gronberg, 2017). Manure estimates were based on animal life span, animal population inventories, and manure N and P content (Gronberg & Arnold, 2017). The approach for adapting the manure and fertilizer estimation methods for years 1982–2012 to our period of record (1950–2017) are documented in Falcone (2020). Wastewater treatment facility data were derived from the U.S. Environmental Protection Agency (USEPA) Clean Watershed Needs Survey (CWNS) data and were aggregated to a watershed scale (Falcone, 2017). Because the CWNS data used here somewhat under-represent total wastewater treatment facility sites (most major dischargers are included but some minor dischargers are not), the effluent estimates given here similarly under-represent the total effluent load (Ivahnenko, 2017). This was the only data source, however, that consistently derived point source loads over multiple decades for the MRB. N-fixation was based on methods established in Sabo et al. (2019), and N-fixation rates were equal to nitrogen uptake and removal in crop harvest (International Plant Nutrition Institute, 2012). N atmospheric deposition estimates were derived from USEPA Critical Loads (US Environmental Protection Agency, 2020). We only included the oxidized forms of N in our balance, since ammonia and organic nitrogen deposition were assumed to be recycled from fertilizer and manure and redeposited during the same year in close proximity to the emission source (Jordan & Weller, 1996; McIsaac et al., 2002; Sprague & Gronberg, 2012). The phosphorus weathering rate was constant (7.16 kg km^−2^ yr^−1^) throughout the period of record and was estimated from soil P concentration data in natural settings (limited anthropogenic disturbance) (Terziotti, 2019). We verified this value by comparing it to average P weathering rates for the MRB derived from a global geodatabase (Hartmann et al., 2014) (Table S3 in Supporting Information S1).

Crop uptake was the primary output and was estimated as yield multiplied by nutrient content. Crop yields per unit area were derived from Census of Agriculture (CoA) data for 12 crops and pasture. Nutrient content (kg N or P crop unit^−1^) was based on literature values (Sprague & Gronberg, 2012) and were held constant throughout the study period. Two denitrification by-products were included as outputs. Agricultural N_2_O flux was estimated as 1% of manure and fertilizer (De Klein et al., 2006), and complete agricultural denitrification (N_2_) was estimated by multiplying the agricultural N_2_O flux by a literature-reported N_2_ to N_2_O ratio of 1.7 (Schlesinger, 2009; Sabo et al., 2019). N emissions from rivers was constant (114 kg km^−2^) throughout the period of record and represented 19% of the estimated N delivered to rivers and streams (Robertson & Saad, 2019, 2021).

We estimated the rate of change in the nutrient balances for two time periods, 1950 to 1985 and 1986 to 2017, using the Theil-Sen slope in the zyp R package (version 0.10–1.1) (Bronaugh, 2009). We used the loess function in the base R package to produce the smoothed nutrient balances. More details about the balance estimation methods are available in Supporting Information S1 Methods—Nutrient Balances, Figures S3–S10, and Tables S2–S4.

### 2.3. Trend Attribution

There were three components related to the water quality trend attribution: (a) estimation of changes in watershed nutrient retention, (b) development of a multiple linear regression model to predict TN and TP load changes over time, and (c) evaluation of potential factors driving water quality nutrient trends at the MRO utilizing counterfactuals. We describe each component in more detail here, and more information about the trend attribution methods for the second and third components are available in Supporting Information S1, Methods—Regression Model and Counterfactual Analysis.

The first component focused on combining river loads and nutrient balances into a single metric, nutrient retention, which indicated the annual amount of nutrient exported in the river relative to the nutrient balance:

(3) Retention=(1−(FN River Load/Nutrient Balance))∗100

Trends in retention from 1975 to 2017 were also estimated using the Theil-Sen slope in the zyp R package (version 0.10–1.1) (Bronaugh, 2009).

The second component focused on the development of a multiple linear regression model to predict TN and TP load changes over time. River loads are a function of many processes, such as time, discharge, and season, as modeled by WRTDS, but also of current nutrient balances, legacy effects from accumulated nutrients in the watershed, and other latent processes difficult to quantify and therefore model, such as BMPs and watershed buffering capacity. To better understand the effect of current and legacy nutrients as well as other potential drivers of long-term changes in river loads, we modeled annual FN river loads as a function of current nutrient balances, lagged nutrient balances, and a latent variable representing an aggregate effect of factors for which we did not have data. Contemporary (current year) nutrient balances, which matched the same year as the river load data, and legacy (historical) N and P balances, which were sequentially shifted in 1-year increments up to a lag of 25 years, were considered as independent variables. For the historical balances, 2017 would use the balances in 1992–2016 for the 1-year through 25-year lags, respectively. Twenty-five-years was the maximum lag, as 1950 was the first year that we had nutrient balance data.

In order to provide more specific guidance on the most relevant time-lagged nutrient balances to include in the model selection process, the N and P FN river load time series were pre-whitened (reduced to white noise) prior to lag correlation analysis by fitting an autoregressive model of order 1 (AR1) (Crawley, 2012). The new pre-whitened series were the residuals from these AR1 models and were used in the lag correlation analysis. We used the “rcorr” function in Hmisc R package (v4.4–2) (Harrell, 2019) to examine the relationship between nutrient balance and FN river loads. For N, only the time-lags with positive and significant correlations (*p*-value < 0.05) to river loads were used to inform which of the 25 time-lagged variables were included in the model selection step. Current (time lag zero) and 1 to 15-year time-lagged N balances were included in the model selection process. For P, only the time-lags with positive correlations to river loads were passed on to the model selection step, and this included current, as well as 1- to 4-year time-lagged P balances (Figure S11 in Supporting Information S1).

The models also included a Year term that served as a latent variable representing the aggregate effects of other factors in the watershed that changed over time but were not captured by the current and lagged nutrient balance terms and could not be quantified directly due to limited data availability. Since river N and P load patterns can be non-monotonic, the options for an independent year variable were represented as first, second, or third degree polynomials. We did not consider a model term for discharge, because FN loads were the dependent variable in the model, and the effects of streamflow on river loads is implicitly accounted for during the flow-normalization process. The relevant current or time-lagged balances from the correlation analysis and the first, second, and third degree polynomial Year terms were considered in the model selection framework, using the “regsubsets” function from leaps R package (version 3.1) (Lumley, 2020). The Bayesian Information Criterion (BIC) was used to select the best model; the lowest BIC value is preferred (Schwarz, 1978) (Figure S12 in Supporting Information S1). Model performance evaluations included a detection of bias in residuals (Figure S13 in Supporting Information S1) and a visual assessment of model fit (Figure S14 in Supporting Information S1).

The third component of our trend attribution was an impact evaluation and was framed using a counterfactual approach, which is a technique that formally compares what actually happened to what would have happened under different conditions (Ferraro, 2009). We examined two different counterfactual scenarios, using hypothetical inputs to the calibrated TN and TP regression models. The original inputs refer to the time-lagged N and P balances and year values used to estimate the predicted river loads outlined in the second component of the Trend Attribution, the regression model. For Counterfactual A, the hypothetical inputs were current and lagged nutrient balances held constant at 1975 levels through 2017, and the Year terms were the same as the original inputs (Tables S6 and S7 in Supporting Information S1). The objective of holding the nutrient balance inputs constant was to investigate how river nutrient loads might have changed between 1975 and 2017 in the absence of any variability in nutrient balances after 1975. For Counterfactual B, the hypothetical inputs were the latent Year term held constant at 1975 levels through 2017, and the current and lagged nutrient balance inputs were the same as in the original inputs (Tables S8 and S9 in Supporting Information S1). The objective of holding the year input constant at 1975 was to investigate how river nutrient loads might have changed between 1975 and 2017 in the absence of any variability in latent processes, potentially accounting for factors such as BMP implementation or watershed buffering capacity. The impact analysis compared the mean annual counterfactual analysis results to the mean original regression results for the time period 2013 to 2017. More details about trend attribution methods are available in Supporting Information S1, Methods—Counterfactual Analysis.

## 3. Results

### 3.1. River Nutrient Load Trends

**Figure 1.**
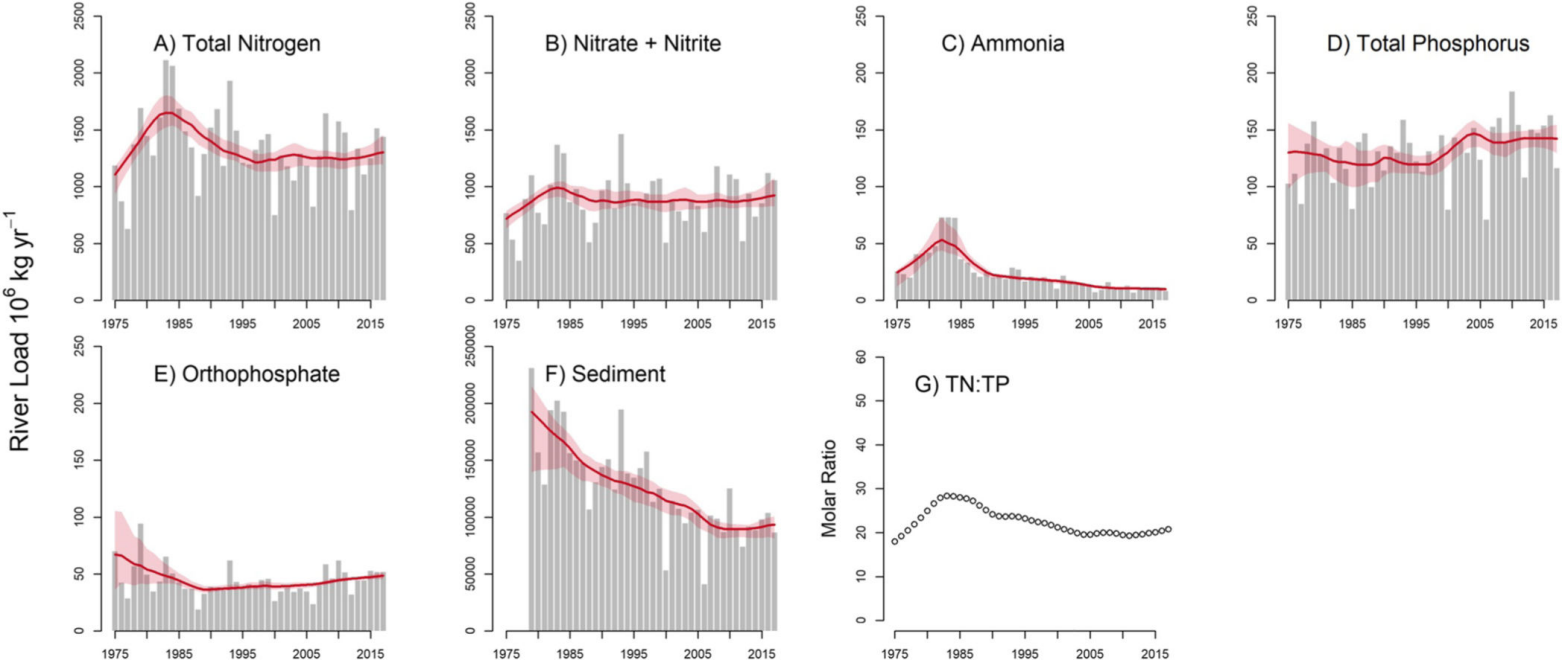
Annual nutrient loads (10^6^ kg yr^−1^) (a–f) and nutrient ratios (g) for the MRO. The gray bars in panels (a–f) are the WRTDS Kalman Filter results. Red line shows the WRTDS flow normalized results and pink shaded area represents the 90% confidence intervals. Input data for each constituent had a start date of October 01, 1974 and an end date of September 30, 2017, with the exception of the Suspended Sediment concentration data, which were available were available from October 01, 1978 to September 30, 2017. Note the difference in the *y*-axis scales among the constituents. Panel (g) shows annual TN:TP molar ratios from 1975 to 2017.

The annual FN river loads for the six water quality constituents were dissimilar. The decade between 1975 and 1985 captured the largest load increases for all three N compounds (TN, NO3, and NH3), but in the subsequent decades the magnitude and trend directions for TN, NO23 as compared to NH3 diverged (Figures 1a–1c and Table 1). Following the 75% increase in NH3 load between 1975 and 1985, the magnitude of the percent load reduction for the subsequent two decades, 1985–1995 and 1995–2005, was large enough to produce a significant decrease in NH3 for the entire study period of record (1975–2017). NH3 only represented 2% of the TN load on average, and the magnitude of change in NH3 loads was not great enough to affect the magnitude or direction of the TN trends. In contrast, on average NO23 represented 66% of TN loads, and the direction and the magnitude of the percent change in the decadal trends were consistent between two N compounds.

The load trend patterns of the two P compounds were different (Figures 1d and 1e, Table 1). Percent changes in TP loads from 1975 to 1995 were minimal, with a sharp increase in TP loads for the time period 1995–2005, but loads remained relatively constant from 2005 to 2015. In contrast, although OP loads increased from 1995 to 2015, the percent decreases in orthophosphate loads from 1975 to 1995 were large enough in magnitude that there was a significant overall downward trend in OP loads for the entire study period. OP only comprised 33% of the TP loads, and decreasing trends in the dissolved P component meant that the particulate P component represented more than 60% of the TP load delivered to the GoM from 1990 onward. Sediment associated transport of nutrients often contributes a significant proportion of both N and P to river systems, and SS loads decreased between 1979 and 2017 (Figure 1f). There were downward trends in both SS and TN from 1980 to 1995, while the annual TP patterns for that same time period did not change. SS loads continued to decrease from 1995 onward without matching decreases in either TN or TP. Because the N and P river load trends were asynchronous, the N:P stoichiometry changed over time. The TN:TP ratio increased between 1975 and 1984, from a low of 18 to a high of 28. Then, the ratio decreased to 20 and remained relatively steady at that level from 2001 to 2017 (Figure 1g).

**Figure 2.**
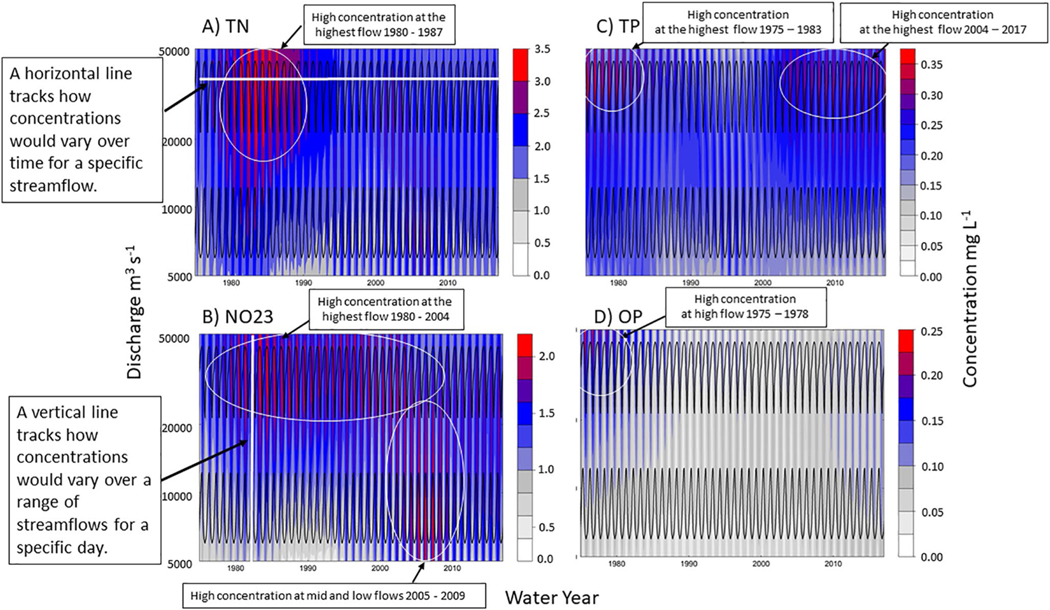
Contour plot showing the changes in concentration-discharge relationships over time for: (a) Total Nitrogen (TN), (b) Nitrate plus Nitrite (NO23), (c) Total Phosphorus (TP), and (d) Orthophosphate (OP) for the MRO. Year, shown on *x*-axes, ranges from 1975 to 2017. Discharge, shown on *y*-axes, ranges from 5,000 to 50,000 m^3^ s^−1^. The upper and lower black lines represent the 5th and 95th daily flow percentiles. The variability in concentrations, shown on the *z*-axes, range from 0 to 3.5 mg L^−1^ depending on the constituent.

Concentration-discharge (C-Q) relationships integrate information about various factors that can affect the river load trends over time, including changes in constituent source, shifts in transport mechanisms, or alternate flowpaths. We highlight the most notable changes in the TN, NO23, TP, and OP concentration-discharge relationships for the MRO site for the time period 1975–2017. The highest TN concentrations (>3.0 mg L^−1^) occurred at the highest flows from 1980 to 1987 (Figure 2a). Continuing to track the horizontal line across the highest streamflows, it is apparent that between 1988 and 2017, the highest TN concentrations decreased to 2.5–3.0 mg L^−1^. From 1980 to 2004, the highest NO23 concentrations (2.0 mg L^−1^) (Figure 2b) also occurred at the highest flows, but the highest NO23 concentrations shifted to mid- and low-flows in 2005, and from 2005 to 2009 the concentrations were highest at the lowest flows. NO23 concentrations at the lowest flows decreased to 1.5 mg L^−1^ from 2010 to 2017. The highest TP concentrations (0.35 mg L^−1^) occurred at the highest flows 1975 to 1983 (Figure 2c), but from 1984 to 2003 the highest concentrations had decreased (0.25 mg L^−1^). However, from 2004 through 2017 the highest TP concentrations (0.30 mg L^−1^) returned at the highest flows. In contrast, the highest orthophosphate concentrations (0.20 mg L^−1^) occurred only at the highest flows from 1975 to 1978 (Figure 2d).

### 3.2. Nutrient Balances

**Figure 3.**
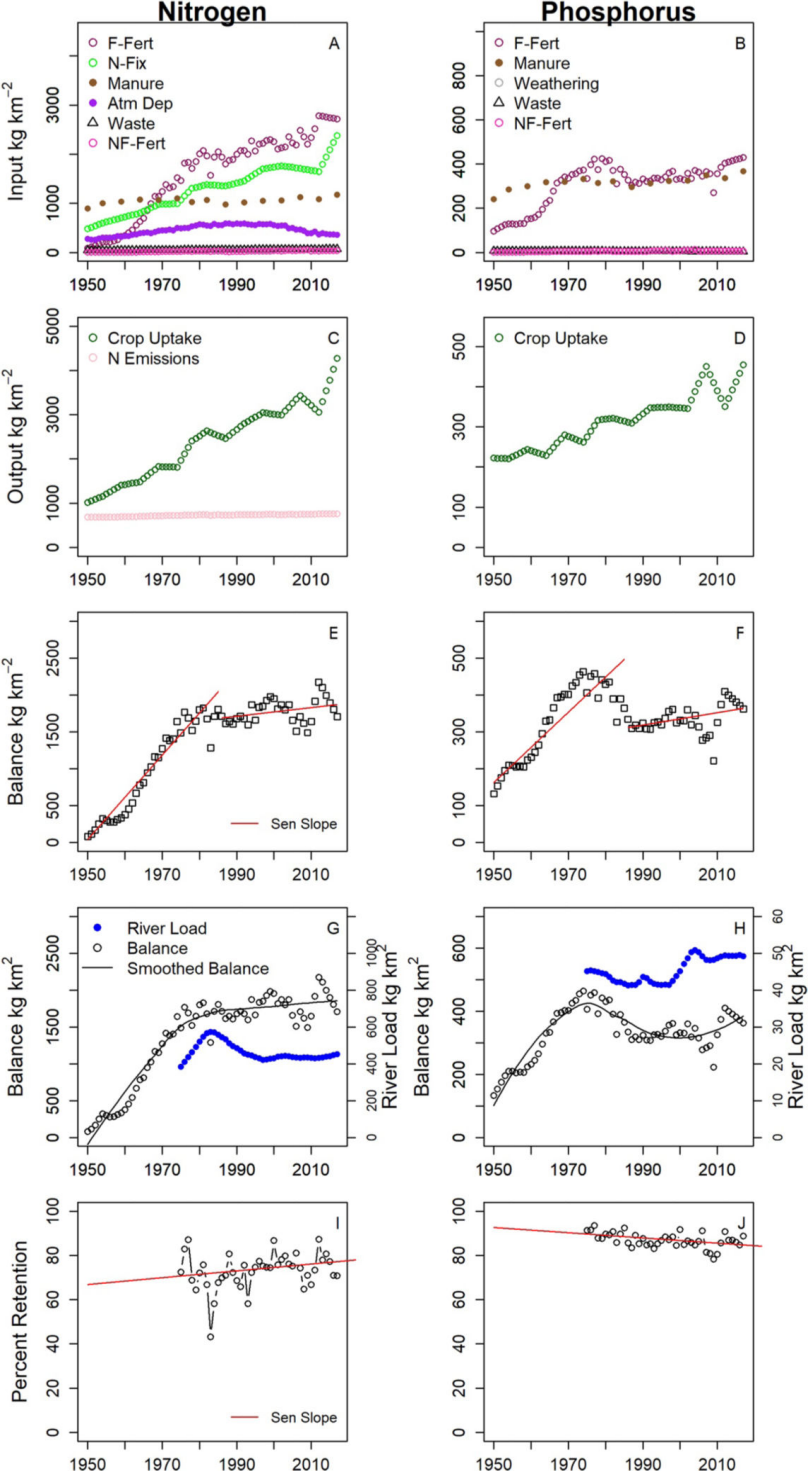
Nutrient inputs (a–b) outputs (c–d), balances (e–f), river loads and balances (g–h), and percent retention (i–j) at the MRO for Nitrogen (left column) and Phosphorus (right column). For panels (a) and (b), F-Fert = Farm Fertilizer, N-fix = N-fixation, Atm Dep = Atmospheric Deposition, Waste = Waste-Water Treatment Facility Effluent, NF-Fert = Non Farm Fertilizer. For panels (e) and (f), Balances = Inputs − Outputs. For panels (i) and (j), Percent Retention (1 – [FN River Load/Nutrient Balance])*100. For panels (e), (f), (i), and (j), Sen Slopes for N and P balances (1950–1985 and 1986 to 2017) and percent retention (1975–2017) are shown. Panels (g) and (h) show the FN WRTDS river loads and the loess smoothed nutrient balances. Note the difference in *y*-axis scales.

There were consistent nutrient surpluses, with average balances of 1,362 and 331 kg km^−2^ yr^−1^ for N and P, respectively, for the time period 1950 to 2017. Farm-fertilizer was the single largest input during the study period, representing 35% of total N inputs and nearly 50% of total P inputs (Figures 3a and 3b; Table S10 in Supporting Information S1). Manure represented 25% of total N inputs and 50% of P inputs. N-fixation was 29% of total N inputs. The proportional contribution of waste-water effluent to the total nutrient inputs was small at <1%, but these were direct and unattenuated inputs into the river. Atmospheric deposition represented 10% of the N inputs, while weathering represented 1% of P inputs. Crop uptake was the largest output for both N and P (Figures 3c and 3d), while losses of N_2_O and N_2_ to the atmosphere represented 25% of N outputs.

Between 1950 and 1985, N and P balances increased at a rate of 2,100 and 340 kg km^−2^ yr^−1^, respectively, but from to 1986 to 2017, the rates of N and P balance increases slowed to 190 and 54 kg km^−2^ yr^−1^, respectively (Figures 3e and 3f). Near the same time as the nutrient balances started to slow down (1986), we also documented a shift in the river load response to nutrient balances. Specifically, from 1975 to 1985 annual nutrient balances and river loads were positively correlated (Figures 3g and 3h). N balances and river loads increased, and P balances and river loads decreased. After 1985, there was a disconnect between both the N and P balances and loads; N balances increased slightly while N loads decreased between 1985 and 1995 and then remained relatively stable through 2017. P balances decreased between 1985 and 1995, remained relatively stable from 1996 to 2005, then increased through 2017, while river P loads were stable between 1985 and 1995, increased between 1995 and 2005, then were stable again through 2017.

### 3.3. Trend Attribution

The attribution of water quality trends had three components: (a) estimation of watershed nutrient retention, (b) multiple linear regression, (c) and counterfactual analysis. Retention reflects the aggregate effects of factors other than changing nutrient balances that may be affecting nutrient loads. Nitrogen retention increased from an average of 70% between 1975 and 1984 to a high of 78% for the decade 1995 to 2004 (Figure 3i). In contrast, the average retention of P declined from approximately 90% between 1975 and 1984 to a low of 85% between 2005 and 2015 (Figure 3j).

The second component of the trend attribution aimed to develop a regression model capable of capturing the simultaneous influence of contemporary nutrient balances (lag0 term), lagged nutrient balances reflecting contributions from legacy nutrients retained in the watershed (higher lag terms), and the aggregate effect of potential latent processes on changing nutrient loads over time (Year terms). The most efficient model explaining TN loads included the Year, Year^2^, Lag2, Lag4, Lag9, and Lag11 terms. The parameterization indicated that legacy nitrogen balances lagged by 11 years or less were contributing to TN loads, and the higher standardized coefficients (Table 2) indicated that longer time lags (Lag9 and Lag11) explained more of the temporal variability in TN loads than shorter time lags (Lag2 and Lag4) (Table 2, Figures S12–S15 in Supporting Information S1); contemporaneous N balances (Lag0) were not significant in the model. The quadratic Year term (Year^2^), captured the different effects of latent processes over time; initially, between 1975 and 1985 there was an increase in the TN loads as the magnitude of the Year terms increased, but after 1995, the relationship changed, and the TN loads decreased.

The most efficient model explaining changes in TP river loads included Year, Year^2^, Year^3^, and Lag4 terms. The P regression model indicated that the Year and the Lag4 terms were the most significant. The parameterization indicated that the time-lag of legacy nutrients which explained a significant amount of variability in TP loads was 4-year; contemporaneous P balances (Lag0), were not significant. The positive linear Year term implied that latent processes had a negative effect on water quality by increasing TP loads. Adding the quadratic term and cubic Year terms captured the different responses of the river TP loads to the increasing Year terms. For years 1975–1985, the latent processes contributed to a decrease in the TP loads, but for years 1995–2005, the increase in TP loads reversed course, and latent processes contributed to increasing TP loads, only to reverse course again after 2005.

**Figure 4.**
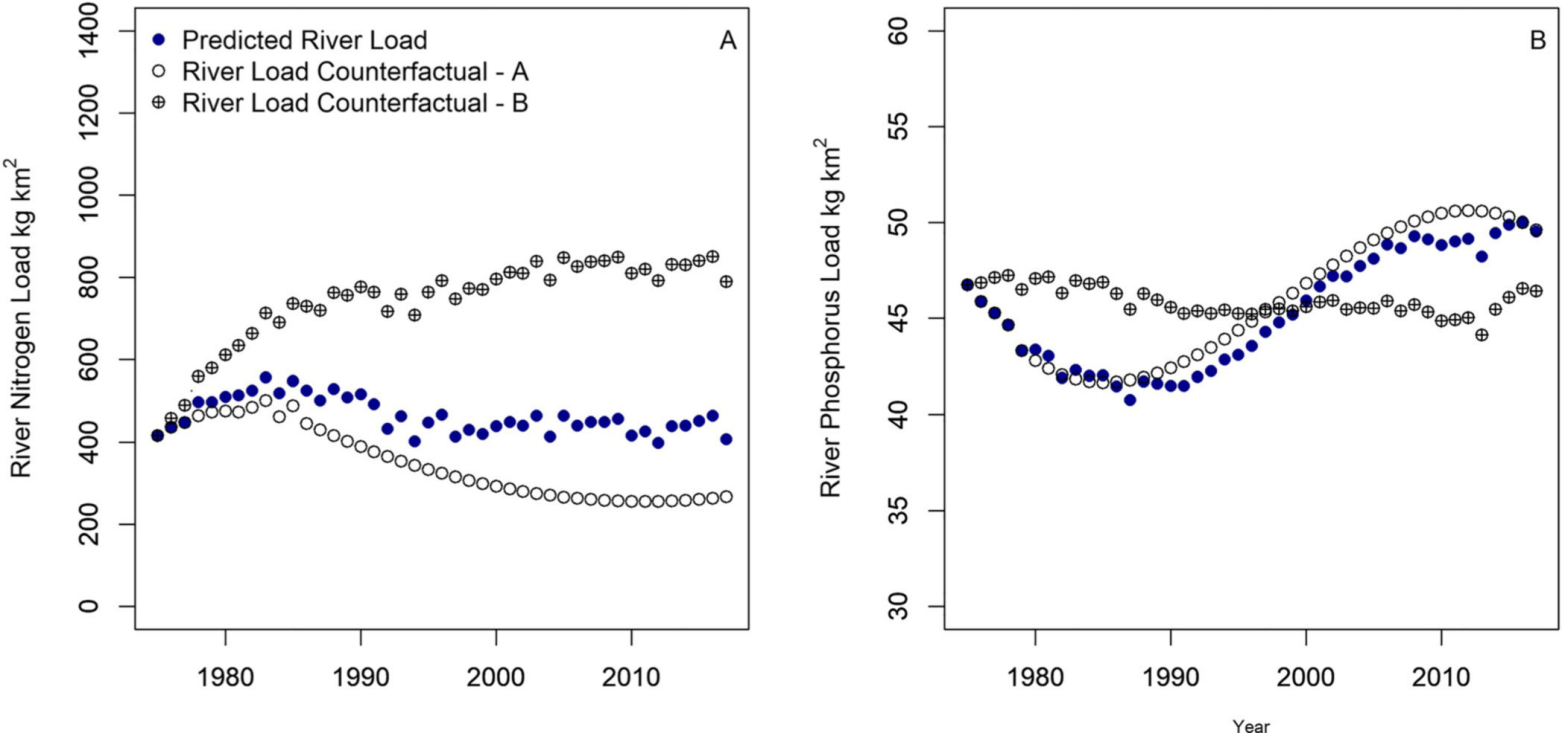
Predicted river loads with regression model are compared to counterfactual results for (a) Total Nitrogen, and (b) Total Phosphorus. Counterfactual A explores the impacts of trends in river loads in the absence of changes in nutrient balances after 1975, and Counterfactual B explores the impacts of trends in river loads in the absence of changes to latent processes.

The third component of trend attribution, the counterfactual analysis, was implemented to test hypotheses about the influence of nutrient balances and latent processes on river loads. The specific objective of Counterfactual A was to investigate how river nutrient loads might have changed between 1975 and 2017 in the absence of any variability in nutrient management after 1975. The N balances from 1964 (time lag 11 years) to 1975 were lower than the balances from 1976 onward; therefore, the magnitude of N balances represented in the Counterfactual A inputs were lower than original inputs, and counterfactual A TN loads for the time period 2013 to 2017 were 50% lower than the original N loads (221 vs. 440 kg km^−2^, Figure 4a). In contrast, river TP loads between 2013 and 2017 were little affected by holding the lagged P balance constant at 1975 levels, and Counterfactual A TP loads (51 kg km^−2^) were marginally higher than the original TP loads of 49 kg km^−2^ (Figure 4b). The small difference between the two sets of results occurred despite P balances from 1971 (time lag 4 years) to 1975 being higher than many of the P balances occurring from 1976 onward.

The primary objective of holding the Year input constant at 1975 in Counterfactual B was to investigate how river nutrient loads might have changed in the absence of any variability in latent processes between 1975 and 2017. The Counterfactual B TN loads were two times higher (910 kg km^−2^) than the original TN loads (440 kg km^−2^, Figure 4a). If latent processes had not been in effect, then TN loads would have doubled. Therefore, this approach indicates that latent processes have been key in decreasing N loads since the mid-1980s. Keeping the three Year terms constant for the TP model had an opposite effect and Counterfactual B TP loads that were slightly lower (46 kg km^−2^) than the original TP loads (49 kg km^−2^, Figure 4b). Although the magnitude of the difference between Counterfactual B and original TP results was only 1 kg km^−2^ greater than the difference between Counterfactual A and original TP results, the pattern of predicted Counterfactual B results was much different than temporal load patterns for Counterfactual A and the Predicted River loads.

## 4. Discussion

### 4.1 River Nutrient Load Trends

Because flow normalized river loads are independent from random variations in streamflow, they are useful for tracking long-term changes in water quality, and for this study, they provided key insights into the progress made to reduce nutrient loads delivered to the GoM. The decreases in both OP and NH3 river loads between 1975 and 2017 and the TN and NO23 load reductions from 1985 to1995 indicate that some water quality improvements have been achieved (Figure 1). Decreasing trends in OP and NH3 corresponded with similar decreases documented in other river basins in the United States, and these widespread dissolved N and P river load reductions were attributed to waste-water treatment facility upgrades and phosphate reductions in laundry and dishwater detergents (Carey & Migliaccio, 2009; Sabo, Clark, Gibbs, et al., 2021; Stets et al., 2020). However, stable TN loads from 1995 to 2017, along with significant increases in TP loads between 1995 and 2005, indicate that water quality challenges remain (Table 1). Additionally, the temporal patterns in the annual FN river loads for the six water quality constituents were asynchronous, suggesting that the environmental controls producing these temporal patterns varied by constituent. For example, sediment-associated transport of nutrients often contributes a significant proportion of both N and P to river systems, and decreases in SS loads are often linked with corresponding decreases in nutrient loads over time (Cohn, 1995). However, that was not the case with our results; while SS loads consistently decreased over the period of study, the temporal trends in TN and TP loads did not show that same pattern. Declines in the MRO SS loads may have been driven by channel improvements, reservoir construction, and soil conservation practices (Keown et al., 1986; Meade & Moody, 2010; Mize et al., 2018), but other factors likely are contributing to N and P trends, confounding the relationship between SS and nutrient loads over time.

Excess nutrients in surface waters stemming from surplus agricultural inputs of N and P on the terrestrial landscape have led to widespread eutrophication in coastal waters (Boesch et al., 2001, 2009; Heisler et al., 2008; Vitousek et al., 2009). The receiving ocean waters in the GoM are nutrient poor, and N has been considered a limiting nutrient on an annual basis, due to efficient recycling of P and denitrification of N (Lohrenz et al., 1997; Turner et al., 2006). However, there is also evidence of P limitation on phytoplankton growth in the spring and summer because of increased river N loads (Bianchi et al., 2010; Sylvan et al., 2006). Nutrient limitation can best be represented with total nutrient forms, like TN and TP, as opposed to dissolved constituents, because the particulate forms account for the N and P assimilated by algae or absorbed by sediment (Bennett et al., 2017; Dodds, 2003). TN:TP molar ratios of less than 20:1 indicate N limitation, ratios that are greater than 50:1 indicate P limitation, and ratios between indicate both N and P limitation (Dzialowski et al., 2005). Past evaluations of TN:TP ratios for the MR from 1974 to 2004 were between 20:1 and 40:1, and recommendations for management were to reduce both river N and P loads to limit eutrophic conditions that lead to hypoxia in the GoM (Turner et al., 2006; Turner & Rabalais, 2013). In our study, the average TN and TP concentrations from 1975 to 2017 were 146 and 7 μmol L^−1^, respectively. These values are well below the thresholds for crossing from mesotrophic to eutrophic conditions in river systems, which have been set at 1,500 and 75 μmol L^−1^, respectively (Dodds & Welch, 2000). However, annual FN TN and TP nutrient river load trends from 1975 to 2017 were asynchronous, leading to shifts in the TN:TP ratios, which could also have implications for eutrophication and organic matter production in the MR and the GoM. We found that the significant increases in TN compared to TP river loads from 1975 to 1985 caused an increase in TN:TP ratio. However, decreases in TN and increases in TP caused a reduction in the TN:TP ratio over time. Despite the shifts, the range of ratios had a range of 18:1 to 28:1, and we infer from those values that both nutrients can limit organic matter production.

Changes over time in concentration-discharge (C-Q) relationships may reflect shifts in the supply of the nutrient source, variations in flow paths, alterations in land-use practices, or major upgrades to waste-water treatment plants to reduce point sources (Hirsch, 2014; Moatar et al., 2017). In our study, there were shifts in the C–Q relationship of both particulate and dissolved N and P (Figure 2) between 1975 and 2017, and here we hypothesize about the relationship between the differences in the C-Q relationships over time and changes within the basin that affect the magnitude of source material or transport of these nutrients into surface waters. The highest TN concentrations (3.5 mg L^−1^) occurred at the highest flows from 1980 to 1987, and this positive C-Q relationship may be associated with flushing of nutrients via surface runoff or erosion (Jordan et al., 1997). By 1988, the highest concentration was 2.5 mg L^−1^ and occurred at mid- to low flows. This shift away from the highest concentrations indicated that the N source transported in runoff may have been reduced or mitigated, and the lower concentration may also indicate dilution of the N source. The highest NO23 concentrations (2.0 mg L^−1^) were also at the highest flows from 1980 to 2004, but the highest NO23 concentrations shifted to low flows from 2005 to 2009, which indicated possible increases in N loading to the MR from groundwater sources (Sprague et al., 2011) (Figure 2b).

Our long-term water quality results show that the highest TP concentrations (0.35 mg L^−1^) occurred at the highest flows from 1980 to 1987 (Figure 2c), but by 1998 the highest concentrations decreased (0.25 mg L^−1^) and the highest concentrations occurred at medium flows. This change in the C-Q relationship would support the theory of a downward shift in surface TP transport, suggesting some progress in managing the amount of P leaving the land surface and reaching rivers during runoff events. This is also consistent with decreased P balances (Figure 3f) and increased no-tillage practices in the MRB (Figure S16 in Supporting Information S1). The limited long-term data available regarding tillage practices show that no-till acreage in the MRB has increased from only approximately 1% of cropland acreage since the 1980s to >40% in 2017, intensive till has decreased from 45% to 30% for the time period 1989 to 1997, and CRP increased from <1% to nearly 10% for the same time period (see Figure S16 and Table S11 in Supporting Information S1) (Baker, 2011; Horowitz et al., 2010). H mg L^−1^) at the highest flows. This indicates a potential reconnection with source material, which enabled a flushing of TP into the river at the highest flows. The highest OP concentrations (0.25 mg L^−1^) occurred at the highest flows from 1975 to 1978, but from 1979 to 2017, the OP concentrations decreased and remained steady at about 0.10 mgL^−1^, which may indicate a reduction in source material, either from agricultural areas because of changes in management practices or from more developed areas because of waste-water treatment facility upgrades or phosphate reductions in laundry and dishwater detergents.

### 4.2 Nutrient Balances

Our study used nutrient balances as one metric to reflect some of the nutrient management practices that have been implemented to reduce surface water pollution from point and nonpoint sources at the MRO. Nutrient inputs, which are a target of many management actions, include: (a) reducing fertilizer and manure application through optimization of application timing, rates, and locations, (b) use of N-fixing crops, (c) reducing atmospheric deposition of nitrogen through decreases in fossil fuel production, (d) reducing wastewater effluent by upgrading treatment facilities, (e) increasing crop yields by efficient use of nutrient amendments and irrigation, and (f) reducing N emissions through improved timing and application of nutrient amendments. A reduction in surplus nutrients (inputs more closely matching outputs over time) reflects the potential success of these collective nutrient management activities. However, not all nutrient management actions, or their potential benefits to water quality, are reflected in the nutrient balances. For example, BMPs like conservation tillage or riparian buffers, which are used to reduce soil erosion and nutrient transport from fields to water bodies are not represented in the changes in nutrient balances over time. Furthermore, although surplus nutrient balances may contribute to an exceedance of a watershed’s buffering capacity, the buffering capacity is not represented in the nutrient balances. However, the effects of both BMPs and changes in watershed buffering capacity may be reflected in changes in either the river N and P river loads or retention over time.

Our methods produced an N balance estimate similar to previous surplus estimates for the MR Basin (David et al., 2010; Hong et al., 2011; McIsaac et al., 2002; Sabo et al., 2019). However, Van Meter et al. (2017) reported a 6,400 kg km^−2^ yr^−1^ surplus for the time period from 1990 to 2014, over four times the surplus N reported in our study. The Van Meter surplus estimate was greater than in this study and others in part because the N-fixation and fertilizer inputs were larger. Our average P balance estimates were much lower than generalized estimates for the United States and Europe (Carpenter et al., 1998), but within the range of agricultural balances reported in Goyette et al. (2018); Sabo, Clark, and Compton et al. (2021); Sabo, Clark, Gibbs, et al. (2021). The consistent N and P surpluses suggest that we have not yet reached the optimal point where nutrient inputs are balanced with nutrient outputs.

Farm-fertilizer was the single largest input during the study period, but previous research indicates that on average only 5%–10% the load from fertilizer ultimately reaches rivers and streams (Robertson & Saad, 2019). Heavier fertilizer use followed increased corn production in response to the demand for more renewable fuels driven by the 2005 Energy Policy Act (Donner & Kucharik, 2008). The decreases in both OP and NH3 river loads between 1975 and 2017 correspond point source reductions (Carey & Migliaccio, 2009; Sabo, Clark, Gibbs, et al., 2021; Stets et al., 2020). Losses of N_2_O and N_2_ to the atmosphere represented 25% of N outputs. Larger losses of N to the atmosphere are reported by other studies (Tian et al., 2020), and this variable remains one of the largest sources of uncertainty in our N balances. Our results indicated that nutrient contributions to MR loads are predominately from nonpoint sources; point source upgrades to wastewater treatment systems may represent a larger proportion of a catchment or watershed-scale nutrient budget, where the nutrient budget is not dominated by agricultural inputs (Robertson & Saad, 2019; Sabo, Clark, & Compton, 2021). Despite consistent nutrient surpluses, there was evidence for improvement, as the rate of increase in nutrient balances slowed in recent decades. The change in the relationship between the river loads and nutrient balances over time (Figures 3g and 3h) indicates that variability in the watershed nutrient balances may have been a primary factor influencing river nutrient loads between 1975 and 1985, but that some other factor or factors became important after 1985.

### 4.3. Trend Attribution

The water quality trend attribution had three components: (a) estimating watershed nutrient retention, (b) multiple linear regression, and (c) counterfactual analysis. Retention reflects the aggregate effects of factors other than changing nutrient balances that may be affecting nutrient loads. Nitrogen retention increased and may have been a factor contributing to lower TN river loads between 1985 and 1995. In contrast, P retention declined, which may have been a factor contributing to higher TP river loads from 1975 to 2017. Increased nutrient N retention may indicate that the watershed buffering capacity had not been met, while reduced P retention may indicate that the watershed buffering capacity may have been exceeded. The effect of changing watershed retention on changing river loads is further explored in the regression modeling and counterfactual impact analyses.

The second component of the trend attribution aimed to develop a regression model. The most efficient model explaining TN loads included Year, Year^2^, Lag2, Lag4, Lag9, and Lag11 terms. Other modeling work (Van Meter et al., 2016) has suggested that it would take >30 years to achieve N load targets to the Gulf of Mexico, even with complete elimination of the N surplus. Because the period of record for our balances was 1950–2017, we were not able to determine the effects of time lags longer than 25 years on our river loads. Our results were in line with recent work, which indicated that the response times of nitrate in 16 intensively managed agricultural catchments with much higher N balances in Brittany, France, was around 2–14 years, and was similar to nitrate time lags for the Chesapeake Bay (Chang et al., 2021; Dupas et al., 2018). The linear and quadratic Year terms explained a significant amount of variability in the river TN loads. The linear Year term had a higher standardized coefficient and was negatively correlated with TN loads, indicating that latent processes overall had an improved effect on water quality by decreasing loads. That finding is consistent with the increased N retention over time, which results in less N export in river loads relative to the nutrient balances over time. The quadratic Year term had a lower standardized coefficient and was positively correlated with TN loads, indicating that there was a change in the relationship between the river N loads and the latent processes over time, which caused an increase in river loads.

The specific objective of Counterfactual A was to investigate how river nutrient loads might have changed between 1975 and 2017 in the absence of any variability in nutrient balances after 1975. These results indicate that targeted efforts to reduce surplus N within the basin could have positive effects on surface water quality, and reductions in TN river loads could potentially be achieved despite N surpluses in the MRB (inputs greater than outputs). The minimal change in Counterfactual A TP loads as compared to original TP loads indicates that changes in P balances may not be very influential on river TP loads. These results further support our conclusions from the TP regression analysis, which showed that the three Year terms and only one lagged balance terms were significant. This suggested that factors other than changing P balances were leading to higher TP river loads over time.

The primary objective of holding the Year input constant at 1975 in Counterfactual B was to investigate how river nutrient loads might have changed in the absence of any variability in latent processes between 1975 and 2017. The Counterfactual B TN loads were two times higher (910 kg km^−2^) than the original TN loads (440 kg km^−2^, Figure 4a). If latent processes had not been in effect, then TN loads may have doubled. Therefore, the latent processes have been key in decreasing N loads since the mid-1980s. Keeping the Year terms constant essentially removed most of the variability in the river TP loads for the time period 1975 to 2017, but the Counterfactual B TP loads for the decade between 1981 and 1990 were higher as compared to the original TP loads, indicating that changes in latent processes may have helped reduce TP loads during this period. For years 2000–2010, the Counterfactual B TP loads were lower than the original TP loads, indicating that changes in latent processes during this period may have contributed to increasing TP loads. As such, latent processes leading to changes in watershed retention had a substantial effect on changing TP loads in the river, but the effect changed from decreasing TP loads to increasing TP loads over the study period.

The model results and counterfactual scenarios indicate that both changing N balances and changing watershed latent processes likely contributed to changes in TN loads. The increase in N balances between 1975 and 2017 contributed to increased TN loads during that period, but these increases were offset by a reduction in the rate of increases in the N balances over time as well as increased N retention in the watershed after the mid-1980s. The model results and counterfactual scenarios indicated that changing P balances likely had a minor influence on changes in TP loads, and changes in latent processes may be much more influential and that the effect of these processes changed from positive (improving water quality and decreasing loads) to negative (degrading water quality and increasing loads).

It is important to note that the Counterfactual B results, which captured latent processes represented by the Year term, may not be entirely independent from the effects of the time-lagged balance terms, which indicated the influence of legacy nutrients and are the focus of Counterfactual A. For example, if the Year term represented BMPs aimed at reducing erosion of nutrient-laden sediments to surface waters, both the legacy and contemporary nutrients on fields may be affected. Also, looking at N specifically, if the Year term represented latent processes that contributed increased buffering capacity through groundwater N storage, then the latent processes are inherently connected to legacy N, since it can take days to years for N to travel from the land surface through groundwater to rivers (Sprague & Gronberg, 2012; Tesoriero et al., 2013).

The Year terms both explained a significant amount of variability in both the TN and TP loads, but the specific latent processes that were captured in the Year term were not identified in the development of the regression models. The results from Counterfactual B, may provide some insight into which latent processes were represented in the Year terms. For both the TN and TP model, we hypothesize that the Year terms may be a proxy for BMPs focused on reducing soil erosion. BMPs, including in-field management (e.g., vegetated buffer strips and cover crops) or edge-of-field practices (e.g., sediment traps or constructed wetlands), reduce physical transfers of nutrients from soils to surface waters (Macintosh et al., 2018). The Year term could also specifically represent changes in tillage practices. Any of the management practices, which were designed to reduce erosion, are a plausible causal factor in explaining MR nutrient load trends. We found significant reductions in SS loads from 1979 to 2017 (Figure 1g), TN and TP concentrations reductions at the highest flows (Figures 2a and 2b), and TN river loads were reduced by 22%, while TP remained unchanged (<1%) during the 1985 to 1995 period as compared to 1975 to 1985. Other regional studies found reductions in N and P loading because of the implementation of BMPs to reduce runoff, including reduced tillage, riparian buffers, grass waterways and terraces (García et al., 2016; Robertson & Saad, 2021). If the Year terms in the TN and TP in the regression model were a proxy for BMPs to reduce erosion, this is significant, because there has been substantial economic investment allocated to the implementation of management practices to reduce loading to freshwater systems by controlling transfer of nutrients at their source in the MRB (Rabotyagov et al., 2014).

Because the Year term is generic and captured a range of latent processes for which we did not have data, we were unable to tease apart the effects of different BMPs. For example, both riparian buffer strips and conservation tillage may reduce erosion and transport of nutrients to surface waters. However, it is important to have a better understanding of both the short- and long-term effects of different management practices, as implementation of no-till practices may initially reduce erosion and transfer of nutrients via surface runoff, but over the long term, the integration of N and P from fertilizer and manure into the soil is reduced, and under no-till, the potential for nitrate to leach beneath the root zone increases because of increased water percolation (McDowell & McGregor, 1984; Power & Schepers, 1989). Our results provide evidence of N sources from groundwater, as there were increased N concentrations at low flows in 2008 and 2009 as compared to before 2007 (Figure 2b). However, this trend was ephemeral, and by 2010 year, the highest concentrations at low flows had subsided. For P, the effects of no-till practices may have initially increased soil retention, reduced erosion, and reduced TP loading, but may have eventually promoted the accumulation and stratification of soil P in upper soil layers (Kleinman et al., 2009; McDowell & McGregor, 1984; Sharpley, 1985) and potentially made P become more readily mobilized and transported during storm and snowmelt events, as P is no longer tilled further down into the soil profile (Kelly et al., 2019). The Year term in the TN and TP models may represent the effects of multiple BMPs to reduce erosion over the large basin. Additionally, the Year term may also have captured the changing effects of different BMPs in the early as compared to the later period of record.

In the TN and TP models, we hypothesize that Year term could also represent changes in the watershed buffering capacity. For example, vegetated buffer strips and sediment traps can be sites for increased retention of N because of reduced erosion, and the increased buffering capacity for N may also be represented in the increased N retention over time. Increased retention of N in groundwater, is another a potential plausible causal factor for variability in river TN loads. Accounting for the differences in excess N storage locations, including groundwater, is important to consider because we found increases in low-flow N concentrations (Figure 2b) and the lag terms (9 and 11 years) were significant in the TN model. This provides some evidence that management may in fact be delaying and not controlling the excess N problem, as the excess N is not transported from fields to streams via surface runoff. Instead, it is routed to groundwater where it can be stored for years to decades. Eventually it reaches the river system and elevates the magnitude of loads. The increase in river TP loads as well as the reduction in retention may also indicate a reduction in the watershed’s ability to absorb additional P. Given the steady surplus of P in the watershed over time it is possible that the terrestrial and hydrologic sinks for surplus P, either in farm soils, riparian areas, stream beds or reservoirs were becoming increasingly saturated (Kleinman, 2017; Zhang et al., 2016). The sinks may have met a threshold above which the buffering was reduced and P was released from storage and exported downstream in river loads (Goyette et al., 2018).

Finally, we hypothesize that the increased riverine P inputs could also be attributed to the effects of enhanced P delivery through tile drains. Field studies have found that enhanced dissolved and particulate P can be transported through subsurface tile drains (Osterholz, Hanrahan, & King, 2020), and the magnitude can increase in soils retaining legacy P. Water discharged through subsurface tile drains passes through the soil matrix, increasing P sorption or assimilation; however, if macropore flow dominates, particularly in fine-textured soils, elevated P concentrations in rivers can result from flow through tile drains (Osterholz, King, et al., 2020), and no-till can enhance macropore development (García et al., 2016; Kleinman et al., 2011). Tile drainage is not routinely captured by agricultural surveys (Jarvie et al., 2017), but a 2012 estimate indicated that the land drained by tile drains is about 5% of the MRB land surface area (Falcone, 2017). Our water quality results only indicted increasing trends for TP; therefore any dissolved P entering the river network through tile drains may have been assimilated into organic matter prior to reaching the MRO (Nemery & Garnier, 2007) (Figures 1d and 1e). One further consideration is that the Year term may also be capturing effects of different management practices in response to climate change effects, specifically those related to heavy precipitation, which has also been found to enhance transfer of nutrients from terrestrial to aquatic systems (Carpenter et al., 2015; Sinha et al., 2017).

### 4.4. Summary and Conclusions

This study utilized 42 years of long-term N and P monitoring data to quantify changes in nutrient trends in both the river and terrestrial landscape environments. We found that nutrient load trends were asynchronous, which suggested that the environmental controls producing these temporal patterns varied for each constituent. Nutrient balances have been considered a key driver of changes in river nutrient loads, and despite consistent surpluses during the study period, we documented that the rate of increase in nutrient balances has slowed in recent decades. This is a key finding, because it shows that nutrient management through an improved alignment of inputs with crop needs has not only slowed the rate of nutrient balance increases, but it also indicates some success in controlling nutrients at the source. Additionally, we documented large decreases in both NH3 and OP, which may indicate the success of waste-water treatment facility improvements and phosphate reductions in laundry and dishwater detergents.

Despite the positive changes related to nutrient balances and reductions in inorganic N and P loads, we did not find evidence of consistent long-term decreases in river TN and TP loads. We did document a large shift in the river load response to changing watershed nutrient balances between 1975 and 2017; annual nutrient balances and river loads were positively correlated between 1975 and 1985, but after, a disconnect between both the N and P balances and river loads emerged, and the subsequent river load trends were different for N versus P. N balances increased slightly while N loads decreased between 1985 and 1995 and then remained relatively stable through 2017. P balances decreased between 1985 and 1995, remained relatively stable from 1996 to 2005, then increased through 2017, while river P loads were stable between 1985 and 1995, increased between 1995 and 2005, then were stable again through 2017.

Time lags in nutrient balances contributed to these patterns, as legacy nutrients on the scale of 2–12 years influenced river load trends. However, we also found that legacy nutrients were not the only factor which may have caused a disconnect between nutrient balances and river loads over time. In the case of N, unmeasured latent processes were just as important to reducing river nutrient loads, and in the case of P, they were even more important. The increase in N balances between 1975 and 2017 contributed to increased TN loads during that period, but these increases were later offset by a reduction in the rate of increases in the N balances over time as well as latest processes after the mid-1980s. If these latent processes had not been in effect, then TN loads may have doubled since the mid-1980s. For P, factors other than changing P balances were leading to higher TP river loads over time; the latent processes had an effect on changing TP loads in the river, and the effect changed from decreasing TP load to increased TP loads over the study period. We did not have data to identify which specific latent process drove the changes in river TN and TP loads, but we considered the potential effects of singular or combined changes in nutrient runoff and transport due to a variety of BMPs, watershed buffering capacity, changes in tillage practices, tile drainage, or increased precipitation. There was evidence that some types of management may in fact be delaying, and not controlling the nutrient problems. We hypothesized that this may be the result of reduced tillage, or no-till practices, which enriched nutrients in surface soils, increasing potential for leaching to groundwater or surface runoff into freshwater ecosystems. This approach, which combines multiple linear regression with counterfactual analysis, shows promise for water quality trends driver analysis, particularly for processes or factors which are not well-represented in typical national scale geospatial datasets. Enhanced datasets and modeling frameworks, which adequately represent the cumulative temporal and spatial effects of the processes and feedbacks stemming from multiple agricultural management practices may be necessary next steps to guide future nutrient management in the MRB.

## Supplementary Material

Supplemental

## Figures and Tables

**Table 1:** Percent Change in Nutrient Fluxes for 10 and 12-Year Increments, the Entire Period of Record (1975–2017), and the Trend Likelihood for the MRO

Compound	1975–1985	1985–1995	1995–2005	2005–2017	1975–2017	1975–2017
						Trend likelihood
TN	+46	−22	< −1	+3.6	+18	Likely up
NO23	+32	−7	−2	+7	+29	Likely up
NH3	+75	−55	−32	−25	−60	Likely down
TP	−8	<−1	+22	−2	+9	Likely up
OP	−34	13	+6	+17	−28	Likely down
SS	NA	−21	−19	−9	−50^a^	Likely down

*Note*. Upward or downward trends were all considered “likely” if the likelihood was >0.85.

a Suspended sediment (SS) trends are for 1979–2017.

**Table 2:** Regression Model Results for Predicting FN TN and TP River Loads for the Time Period 1975 to 2017 for the MRO

	Estimate	SE	*T*-value	*p*-value	Standard coefficient	VIF
Nitrogen model						
Intercept	−524	115	−4.56	<0.0001		
Balance Lag 2 years	0.11	0.03	4.07	0.0002	0.43	1.71
Balance Lag 4 years	0.11	0.03	3.99	0.0003	0.41	1.68
Balance Lag 9 years	0.16	0.03	4.91	<0.0001	0.83	4.38
Balance Lag 11 years	0.18	0.03	5.77	<0.0001	1.11	5.76
Year	−9.1	0.87	−10.4	<0.0001	−2.35	7.82
Year^2^	0.30	0.05	6.45	<0.0001	0.87	2.82
Phosphorus model						
Intercept	39.63	2.048	19.35	<0.0001		
Balance Lag 4 years	0.0128	0.006	2.09	0.04	0.222	2.75
Year	0.4801	0.050	9.6	<0.0001	1.821	8.72
Year^2^	0.0073	0.002	3.82	0.0004	0.307	1.57
Year^3^	−0.0009	0.0001	−6.42	<0.0001	−1.055	6.58

*Note*. The N model *R*^2^ = 0.76 and the P model *R*^2^ = 0.84. VIF, variance inflation factor.

## Data Availability

Additional information about methods and results are provided in the associated Supplemental Information file. The river loads and nutrient balance data used to generate results in this manuscript are all available in a related data release see Stackpoole et al. (2021).

## Key Points:

- Some positive water quality results were documented, but there is still room for improvement
- The water quality improvements may be the result of successful implementation of long-term nutrient management efforts in the Basin
- Both N and P limit the development of hypoxia in the Gulf of Mexico, but factors that influence the river loads are different, so management will have to address each nutrient separately

## References

[R1] Alexander RB, Smith RA, Schwarz GE, Boyer EW, Nolan JV, & Brakebill JW (2008). Differences in phosphorus and nitrogen delivery to the Gulf of Mexico from the Mississippi River Basin. Environmental Science & Technology, 42, 822–830. 10.1021/es0716103 18323108

[R2] Baker NT (2011). Tillage practices in the conterminous United States, 1989–2004-datasets aggregated by watershed. U.S. Geological Survey Data Series (Vol. 573, p. 13).

[R3] Bennett MG, Schofield KA, Lee SS, & Norton SB (2017). Response of chlorophyll a to total nitrogen and total phosphorus concentrations in lotic ecosystems: A systematic review protocol. Environmental Evidence, 6, 1–13. 10.1186/s13750-017-0097-8 31019679 PMC6475917

[R4] Bianchi TS, DiMarco SF, Cowan JH, Hetland RD, Chapman P, Day JW, & Allison MA (2010). The science of hypoxia in the Northern Gulf of Mexico: A review. The Science of the Total Environment, 408, 1471–1484. 10.1016/j.scitotenv.2009.11.047 20092873

[R5] Boesch DF, Boynton WR, Crowder LB, Diaz RJ, Howarth RW, Mee LD, (2009). Nutrient enrichment drives Gulf of Mexico hypoxia. Eos, Transactions American Geophysical Union, 90, 117–118. 10.1029/2009eo140001

[R6] Boesch DF, Brinsfield RB, & Magnien RE (2001). Chesapeake bay eutrophication: Scientific understanding, ecosystem restoration, and challenges for agriculture. Journal of Environmental Quality, 30, 303–320. 10.2134/jeq2001.302303x 11285890

[R7] Brakebill JW & Gronberg JM (2017). County-level estimates of nitrogen and phosphorus from commercial fertilizer for the conterminous United States, 1987–2012. U.S. Geological Survey data release. 10.5066/F7H41PKX

[R8] Bronaugh D. (2009). zyp: Zhang + Yue-Pilon trends package. Version 0.10–1.1. Retrieved from http://CRAN.R-project.org/package=zyp

[R9] Carey RO, & Migliaccio KW (2009). Contribution of wastewater treatment plant effluents to nutrient dynamics in aquatic systems: A review. Environmental Management, 44, 205–217. 10.1007/s00267-009-9309-5 19458999

[R10] Carpenter SR, Booth EG, Kucharik CJ, & Lathrop RCJAS (2015). Extreme daily loads: Role in annual phosphorus input to a north temperate lake. Aquatic Sciences, 77, 71–79. 10.1007/s00027-014-0364-5

[R11] Carpenter SR, Caraco NF, Correll DL, Howarth RW, Sharpley AN, & Smith VH (1998). Nonpoint pollution of surface waters with phosphorus and nitrogen. Ecological Applications, 8, 559–568. 10.1890/1051-0761(1998)008[0559:nposww]2.0.co;2

[R12] Chang SY, Zhang Q, Byrnes DK, Basu NB, & Van Meter KJ (2021). Chesapeake legacies: The importance of legacy nitrogen to improving Chesapeake Bay water quality. Environmental Research Letters, 16, 085002. 10.1088/1748-9326/ac0d7b

[R13] Cohn TA (1995). Recent advances in statistical methods for the estimation of sediment and nutrient transport in rivers. Reviews of Geophysics, 33, 1117–1123. 10.1029/95rg00292

[R14] Crawley MJ (2012). The R book. John Wiley & Sons.

[R15] David MB, Drinkwater LE, & McIsaac GF (2010). Sources of Nitrate Yields in the Mississippi River Basin. Journal of Environmental Quality, 39, 1657–1667. 10.2134/jeq2010.0115 21043271

[R16] De Klein C, Novoa RS, Ogle S, Smith KA, Rochette P, Wirth TC, (2006). N2O emissions from managed soils, and CO2 emissions from lime and urea application. IPCC guidelines for National greenhouse gas inventories (Vol. 4, pp. 1–54). Prepared by the National greenhouse gas inventories programme. https://www.ipcc-nggip.iges.or.jp/public/2006gl/pdf/4_Volume4/V4_11_Ch11_N2O&CO2.pdf

[R17] Dodds WK (2003). Misuse of inorganic N and soluble reactive P concentrations to indicate nutrient status of surface waters. Journal of the North American Benthological Society, 22, 171–181. 10.2307/1467990

[R18] Dodds WKK, & Welch EB (2000). Establishing nutrient criteria in streams. Journal of the North American Benthological Society, 19, 186–196. 10.2307/1468291

[R19] Donner SD, & Kucharik CJ (2008). Corn-based ethanol production compromises goal of reducing nitrogen export by the Mississippi River. Proceedings of the National Academy of Sciences, 105, 4513–4518. 10.1073/pnas.0708300105 PMC2393748 18332435

[R20] Doody DG, Withers PJ, Dils RM, McDowell RW, Smith V, McElarney YR, (2016). Optimizing land use for the delivery of catchment ecosystem services. Frontiers in Ecology and the Environment, 14, 325–332. 10.1002/fee.1296

[R21] Dupas R, Minaudo C, Gruau G, Ruiz L, & Gascuel-Odoux C. (2018). Multidecadal trajectory of riverine nitrogen and phosphorus dynamics in rural catchments. Water Resources Research, 54, 5327–5340. 10.1029/2018wr022905

[R22] Dzialowski AR, Wang S-H, Lim N-C, Spotts WW, & Huggins DG (2005). Nutrient limitation of phytoplankton growth in central plains reservoirs, USA. Journal of Plankton Research, 27, 587–595. 10.1093/plankt/fbi034

[R23] Falcone JA (2017). Watershed characteristics for study sites of the Surface Water Trends project. National Water Quality Program: U.S. Geological Survey data release. 10.5066/F7TX3CKP

[R24] Falcone JA (2020). Estimates of county-level nitrogen and phosphorus from fertilizer and manure for approximately five-year periods from 1950 to 2017 for the conterminous United States: U.S. Geological Survey Open File Report. 10.3133/ofr20201153

[R25] Ferraro PJ (2009). Counterfactual thinking and impact evaluation in environmental policy. New Directions for Evaluation, 2009, 75–84. 10.1002/ev.297

[R26] Fraterrigo JM, & Downing JA (2008). The influence of land use on lake nutrients varies with watershed transport capacity. Ecosystems, 11, 1021–1034. 10.1007/s10021-008-9176-6

[R27] García AM, Alexander RB, Arnold JG, Norfleet L, White MJ, Robertson DM, & Schwarz G. (2016). Regional effects of agricultural conservation practices on nutrient transport in the Upper Mississippi River Basin. Environmental Science & Technology, 50, 6991–7000. 27243625 10.1021/acs.est.5b03543

[R28] Goolsby DA, Battaglin WA, Lawrence GB, Artz RS, Aulenbach BT, Hooper RP, (1999). Flux and sources of nutrients in the Mississippi-Atchafalaya River basin. National Oceanic and Atmospheric Administration National Ocean Service. https://repository.library.noaa.gov/view/noaa/21437

[R29] Goyette JO, Bennett EM, & Maranger R. (2018). Low buffering capacity and slow recovery of anthropogenic phosphorus pollution in watersheds. Nature Geoscience, 11, 921–925. 10.1038/s41561-018-0238-x

[R30] Gronberg JAM, & Arnold T. (2017). County-level estimates of nitrogen and phosphorus from animal manure for the conterminous United States, 2007 and 2012, USGS Open File Report 2017–1021. 10.3133/ofr20171021

[R31] Harrell FE (2019). Package ‘hmisc’ (pp. 235–236). Retrieved from https://cran.r-project.org/web/packages/Hmisc/Hmisc.pdf.CRAN20182019

[R32] Hartmann J, Moosdorf N, Lauerwald R, Hinderer M, & West AJ (2014). Global chemical weathering and associated P-release—The role of lithology, temperature and soil properties. Chemical Geology, 363, 145–163. 10.1016/j.chemgeo.2013.10.025

[R33] Haygarth PM, Jarvie HP, Powers SM, Sharpley AN, Elser JJ, Shen J, (2014). Sustainable phosphorus management and the need for a long-term perspective: The legacy hypothesis. ACS Publications. 10.1021/es502852s 25001016

[R34] Heisler J, Glibert PM, Burkholder JM, Anderson DM, Cochlan W, Dennison WC, (2008). Eutrophication and harmful algal blooms: A scientific consensus. Harmful Algae, 8, 3–13. 10.1016/j.hal.2008.08.006 28781587 PMC5543702

[R35] Hirsch RM (2014). Large biases in regression-based constituent flux estimates: Causes and diagnostic tools. JAWRA Journal of the American Water Resources Association, 50, 1401–1424. 10.1111/jawr.12195

[R36] Hirsch RM, Archfield SA, & De Cicco LA (2015). A bootstrap method for estimating uncertainty of water quality trends. Environmental Modelling & Software, 73, 148–166. 10.1016/j.envsoft.2015.07.017

[R37] Hirsch RM, Moyer DL, & Archfield SA (2010). Weighted regressions on time, discharge, and season (WRTDS), with an application to Chesapeake Bay river inputs 1. JAWRA Journal of the American Water Resources Association, 46, 857–880. 10.1111/j.1752-1688.2010.00482.x 22457569 PMC3307614

[R38] Hong B, Swaney DP, & Howarth RW (2011). A toolbox for calculating net anthropogenic nitrogen inputs (NANI). Environmental Modelling & Software, 26, 623–633. 10.1016/j.envsoft.2010.11.012

[R39] Horowitz JK, Ebel RM, & Ueda K. (2010). No-till farming is a growing practice. United States Department of Agriculture. Economic Research Service. Economic Information Bulletin Number 70. 10.22004/ag.econ.96636

[R40] International Plant Nutrition Institute. (2012). A Nutrient Use Information System (NuGIS) for the U.S. Norcross, GA. Retrieved from www.ipni.net/nugis

[R41] Ivahnenko TI (2017). Evaluation and use of US environmental protection agency clean watersheds needs survey data to quantify nutrient loads to surface water, 1978–2012. 2328–0328. US Geological Survey Scientific Investigations Report 5115. 10.3133/sir20175115

[R42] Jarvie HP, Johnson LT, Sharpley AN, Smith DR, Baker DB, Bruulsema TW, & Confesor R. (2017). Increased soluble phosphorus loads to Lake Erie: Unintended consequences of conservation practices? Journal of Environmental Quality, 46, 123–132. 10.2134/jeq2016.07.0248 28177409

[R43] Jordan TE, Correll DL, & Weller DE (1997). Relating nutrient discharges from watersheds to land use and streamflow variability. Water Resources Research, 33, 2579–2590. 10.1029/97wr02005

[R44] Jordan TE, & Weller DE (1996). Human contributions to terrestrial nitrogen flux. BioScience, 46, 655–664. 10.2307/1312895

[R45] Kelly PT, Renwick WH, Knoll L, & Vanni MJ (2019). Stream nitrogen and phosphorus loads are differentially affected by storm events and the difference may be exacerbated by conservation tillage. Environmental Science & Technology, 53, 5613–5621. 10.1021/acs.est.8b05152 30861345

[R46] Keown MP, Dardeau EA Jr, & Causey EM (1986). Historic Trends in the Sediment Flow Regime of the Mississippi River. Water Resources Research, 22, 1555–1564. 10.1029/wr022i011p01555

[R47] Kleinman PJ, Sharpley AN, Saporito LS, Buda AR, & Bryant RB (2009). Application of manure to no-till soils: Phosphorus losses by sub-surface and surface pathways. Nutrient Cycling in Agroecosystems, 84, 215–227. 10.1007/s10705-008-9238-3

[R48] Kleinman PJA (2017). The Persistent Environmental Relevance of Soil Phosphorus Sorption Saturation. Current Pollution Reports, 3, 141–150. 10.1007/s40726-017-0058-4

[R49] Kleinman PJA, Sharpley AN, Buda AR, McDowell RW, & Allen AL (2011). Soil controls of phosphorus in runoff: Management barriers and opportunities. Canadian Journal of Soil Science, 91, 329–338. 10.4141/cjss09106

[R50] Lohrenz SE, Fahnenstiel GL, Redalje DG, Lang GA, Chen X, & Dagg MJ (1997). Variations in primary production of northern Gulf of Mexico continental shelf waters linked to nutrient inputs from the Mississippi River. Marine Ecology Progress Series, 155, 45–54. 10.3354/meps155045

[R51] Lohrenz SE, Redalje DG, Cai W-J, Acker J, & Dagg M. (2008). A retrospective analysis of nutrients and phytoplankton productivity in the Mississippi River plume. Continental Shelf Research, 28, 1466–1475. 10.1016/j.csr.2007.06.019

[R52] Lumley T. (2020). Package “leaps”. version 3.1. Retrieved from https://cran.r-project.org/web/packages/leaps/leaps.pdf

[R53] Macintosh KA, Mayer BK, McDowell RW, Powers SM, Baker LA, Boyer TH, & Rittmann BE (2018). Managing diffuse phosphorus at the source versus at the sink. Environmental Science & Technology, 52, 11995–12009. 10.1021/acs.est.8b01143 30247882

[R54] McDowell LL, & McGregor KC (1984). Plant nutrient losses in runoff from conservation tillage corn. Soil and Tillage Research, 4, 79–91. 10.1016/0167-1987(84)90018-7

[R55] McIsaac GF, David MB, Gertner GZ, & Goolsby DA (2002). Relating net nitrogen input in the Mississippi River Basin to nitrate flux in the Lower Mississippi River. Journal of Environmental Quality, 31, 1610–1622. 10.2134/jeq2002.1610 12371178

[R56] Meade RH, & Moody JA (2010). Causes for the decline of suspended-sediment discharge in the Mississippi River system, 1940–2007. Hydrological Processes, 24, 35–49. 10.1002/hyp.7477

[R57] Mize SV, Murphy JC, Diehl TH, & Demcheck DK (2018). Suspended-sediment concentrations and loads in the lower Mississippi and Atchafalaya rivers decreased by half between 1980 and 2015. Journal of Hydrology, 564, 1–11. 10.1016/j.jhydrol.2018.05.068

[R58] Moatar F, Abbott BW, Minaudo C, Curie F, & Pinay G. (2017). Elemental properties, hydrology, and biology interact to shape concentration-discharge curves for carbon, nutrients, sediment, and major ions. Water Resources Research, 53, 1270–1287. 10.1002/2016wr019635

[R59] Murphy J, Hirsch RM, & Sprague LA (2013). Nitrate in the Mississippi River and its tributaries, 1980–2010: An update. US Geological Survey Scientific Investigations Report 5169. https://pubs.usgs.gov/sir/2013/5169/pdf/sir20135169.pdf

[R60] Nemery J, & Garnier J. (2007). Origin and fate of phosphorus in the Seine watershed (France): Agricultural and hydrographic P budgets. Journal of Geophysical Research, 112. 10.1029/2006jg000331

[R61] Osterholz W, King K, Williams M, Hanrahan B, & Duncan E. (2020). Stratified Soil Sampling Improves Predictions of P Concentration in Surface Runoff and Tile Discharge. Soil Systems, 4, 67. 10.3390/soilsystems4040067

[R62] Osterholz WR, Hanrahan BR, & King KW (2020). Legacy phosphorus concentration–discharge relationships in surface runoff and tile drainage from Ohio crop fields. Journal of Environmental Quality, 49, 675–687. 10.1002/jeq2.20070 33016383

[R63] Power JF, & Schepers JS (1989). Nitrate contamination of groundwater in North America. Agriculture, Ecosystems & Environment, 26, 165–187. 10.1016/0167-8809(89)90012-1

[R64] Powers SM, Bruulsema TW, Burt TP, Chan NI, Elser JJ, Haygarth PM, (2016). Long-term accumulation and transport of anthropogenic phosphorus in three river basins. Nature Geoscience, 9, 353–356. 10.1038/ngeo2693

[R65] Rabotyagov SS, Campbell TD, White M, Arnold JG, Atwood J, Norfleet ML, (2014). Cost-effective targeting of conservation investments to reduce the northern Gulf of Mexico hypoxic zone. Proceedings of the National Academy of Sciences, 111, 18530–18535. 10.1073/pnas.1405837111 PMC4284528 25512489

[R66] Robertson DM, & Saad DA (2019). Spatially Referenced Models of Streamflow and Nitrogen, Phosphorus, and Suspended-Sediment Loads in Streams of the Midwestern United States. U.S. Geological Survey Scientific Investigations Report Report 2019–5114. 10.3133/sir20195114

[R67] Robertson DM, & Saad DA (2021). Nitrogen and phosphorus sources and delivery from the Mississippi/Atchafalaya River basin: An update using 2012 SPARROW models. JAWRA Journal of the American Water Resources Association. 10.1111/1752-1688.12905

[R68] Sabo RD, Clark CM, Bash J, Sobota D, Cooter E, Dobrowolski JP, (2019). Decadal shift in nitrogen inputs and fluxes across the contiguous United States: 2002–2012. Journal of Geophysical Research: Biogeosciences, 124, 3104–3124. 10.1029/2019jg005110 PMC12180915 40547514

[R69] Sabo RD, Clark CM, & Compton JE (2021). Considerations when using nutrient inventories to prioritize water quality improvement efforts across the US. Environmental Research Communications, 3, 045005. 10.1088/2515-7620/abf296 PMC9709726 36457483

[R70] Sabo RD, Clark CM, Gibbs DA, Metson G, Todd MJ, LeDuc SD, (2021). Phosphorus Inventory for the Conterminous United States (2002–2012). Journal of Geophysical Research: Biogeosciences, 126, e2020JG005684. 10.1029/2020JG005684 PMC10116864 37089664

[R71] Scavia D, & Donnelly KA (2007). Reassessing Hypoxia Forecasts for the Gulf of Mexico. Environmental Science & Technology, 41, 8111–8117. 10.1021/es0714235 18186345

[R72] Schlesinger WH (2009). On the fate of anthropogenic nitrogen. Proceedings of the National Academy of Sciences, 106, 203–208. 10.1073/pnas.0810193105 PMC2613040 19118195

[R73] Schwarz G. (1978). Estimating the dimension of a model. Annals of Statistics, 6, 461–464. 10.1214/aos/1176344136

[R74] Sharpley AN (1985). The Selection Erosion of Plant Nutrients in Runoff. Soil Science Society of America Journal, 49, 1527–1534. 10.2136/sssaj1985.03615995004900060039x

[R75] Sinha E, Michalak AM, & Balaji V. (2017). Eutrophication will increase during the 21st century as a result of precipitation changes. Science, 357, 405–408. 10.1126/science.aan2409 28751610

[R76] Sprague LA, & Gronberg JAM (2012). Relating Management Practices and Nutrient Export in Agricultural Watersheds of the United States. Journal of Environmental Quality, 41, 1939–1950. 10.2134/jeq2012.0073 23128751

[R77] Sprague LA, Hirsch RM, & Aulenbach BT (2011). Nitrate in the Mississippi River and Its Tributaries, 1980 to 2008: Are We Making Progress? Environmental Science & Technology, 45, 7209–7216. 10.1021/es201221s 21823673 PMC3169996

[R78] Stackpoole SM, Sabo RD, & Falcone JA (2021). Nutrient balances, river loads, and a counterfactual analysis to determine drivers of Mississippi River nitrogen and phosphorus loads between 1975 and 2017. US Geological Survey data release. 10.5066/P9ZM964O

[R79] Stackpoole SM, Stets EG, & Sprague LA (2019). Variable impacts of contemporary versus legacy agricultural phosphorus on US river water quality. Proceedings of the National Academy of Sciences, 116, 20562–20567. 10.1073/pnas.1903226116 PMC6789928 31548416

[R80] Stets EG, Sprague LA, Oelsner GP, Johnson HM, Murphy JC, Ryberg K, . (2020). Landscape drivers of dynamic change in water quality of U.S. Rivers. Environmental Science & Technology, 54, 4336–4343. 10.1021/acs.est.9b05344 32216285

[R81] Sylvan JB, Dortch Q, Nelson DM, Maier Brown AF, Morrison W, & Ammerman JW (2006). Phosphorus limits phytoplankton growth on the Louisiana Shelf during the period of hypoxia formation. Environmental Science & Technology, 40, 7548–7553. 10.1021/es061417t 17256493

[R82] Terziotti S. (2019). Distribution of phosphorus in soils and aggregated within geologic mapping units, conterminous United States. U.S. Geological Survey data release. 10.5066/P918DF1E

[R83] Tesoriero AJ, Duff JH, Saad DA, Spahr NE, & Wolock DM (2013). Vulnerability of streams to legacy nitrate sources. Environmental Science & Technology, 47, 3623–3629. 10.1021/es305026x 23530900

[R84] Tian H, Xu R, Canadell JG, Thompson RL, Winiwarter W, Suntharalingam P, (2020). A comprehensive quantification of global nitrous oxide sources and sinks. Nature, 586, 248–256. 10.1038/s41586-020-2780-0 33028999

[R85] Turner RE, & Rabalais NN (2003). Linking landscape and water quality in the Mississippi River Basin for 200 years. BioScience, 53, 563–572. https://academic.oup.com/bioscience/article/53/6/563/224739

[R86] Turner RE, & Rabalais NN (2013). Nitrogen and phosphorus phytoplankton growth limitation in the northern Gulf of Mexico. Aquatic Microbial Ecology, 68, 159–169. 10.3354/ame01607

[R87] Turner RE, Rabalais NN, & Justic D. (2006). Predicting summer hypoxia in the northern Gulf of Mexico: Riverine N, P, and Si loading. Marine Pollution Bulletin, 52, 139–148. 10.1016/j.marpolbul.2005.08.012 16212987

[R88] United States Department of Agriculture. (2012). Assessment of the effects of conservation practices on cultivated cropland in the Upper Mississippi River basin. Conservation Effects Assessment Project. USDA Nat. Resour. Conserv. Serv. https://www.nrcs.usda.gov/Internet/FSE_DOCUMENTS/stelprdb1042093.pdf

[R89] United States Department of Agriculture. (2013). Assessment of the effects of conservation practices on cultivated cropland in the Lower Mississippi River basin. Conservation Effects Assessment Project. USDA Nat. Resour. Conserv. Serv. https://www.nrcs.usda.gov/Internet/FSE_DOCUMENTS/stelprdb1176978.pdf

[R90] US Environmental Protection Agency. (2020). Critical loads mapper tool. Retrieved from https://www.epa.gov/air-research/critical-loads-mapper-tool

[R91] Van Meter KJ, Basu NB, & Van Cappellen P. (2017). Two centuries of nitrogen dynamics: Legacy sources and sinks in the Mississippi and Susquehanna River Basins. Global Biogeochemical Cycles, 31, 2–23. 10.1002/2016gb005498

[R92] Van Meter KJ, Basu NB, Veenstra JJ, & Burras CL (2016). The nitrogen legacy: Emerging evidence of nitrogen accumulation in anthropogenic landscapes. Environmental Research Letters, 11, 035014. 10.1088/1748-9326/11/3/035014

[R93] Vitousek PM, Naylor R, Crews T, David MB, Drinkwater LE, Holland E, (2009). Nutrient imbalances in agricultural development. Science, 324, 1519–1520. 10.1126/science.1170261 19541981

[R94] Zhang Q, & Hirsch RM (2019). River water-quality concentration and flux estimation can be improved by accounting for serial correlation through an autoregressive model. Water Resources Research, 55, 9705–9723. 10.1029/2019wr025338

[R95] Zhang Q, Hirsch RM, & Ball WP (2016). Long-term changes in sediment and nutrient delivery from conowingo dam to Chesapeake Bay: Effects of reservoir sedimentation. Environmental Science & Technology, 50, 1877–1886. 10.1021/acs.est.5b04073 26744776

