# Supplementary material for: Long-Term Mississippi River Trends Expose Shifts in the River Load Response to Watershed Nutrient Balances Between 1975 and 2017
Source: Water Resour Res. Author manuscript; Available in PMC 2023 Mar 3. (PMC9983731; doi:10.1029/2021wr030318)
Supplement: Supplemental [file NIHMS1809137-supplement-Supplemental.pdf]

Water Resources Research

Supporting Information for

Long-term Mississippi River trends expose shifts in the river load response to watershed nutrient balances between 1975 and 2017

Sarah Stackpoole[1], Robert Sabo[2], James Falcone[3], Lori A. Sprague[4]

[1] USGS,[2] USEPA, [3] USGS,[4] USGS

Contents of this file

Section 1 – Site Information

Section 2 – Methods

River loads

Nutrient Balances

Inputs

Outputs

Trend Attribution

Regression model

Counterfactual analysis

Section 3 – Results

Nutrient Balances

Trend Attribution

Regression model

Section 4 – Supporting data on tillage practices for the Mississippi River Basin

Section 5 - References

Figures S1 – S16.

Tables S1 to S11.

Section 1 – Site Information

Site Information Mississippi River Outflow (MRO) This study reports the water quality trends and nutrient balances for one site on the Mississippi River, which we refer to as the Mississippi River Outflow (MRO) (Figure S1) (Sprague et al. 2011, Murphy et al. 2013). This site is located approximately 225 river miles upstream from the outflow to the Gulf of Mexico. The surface water quality monitoring data used to estimate loads for the MRO were collected from the Mississippi River at St. Francisville, LA, which is located downstream from the controlled diversion systems called the Old River Low Sill and Overbank Structure (USGS Site Number= 07373420, Station Abbreviation= STFR, Latitude = 30.75852, Longitude= -91.39595). This sampling location is downstream from the Old River Outflow Channel, but nutrient concentrations are similar between this location and the location upstream of the Old River Outflow Channel (Coupe et al. 2013) and the location downstream in the delta at the confluence of the Mississippi River with the Gulf of Mexico (Turner et al. 2006). The discharge monitoring data used to estimate loads for the MRO were collected from two US Army Corps of Engineers sites: the Mississippi River at Tarbert Landing (USACE, Site Number = 00110, Latitude=31.00808830, Longitude=-91.62361110) and the Old River Outflow Channel (USACE Site Number = 02600, Latitude=31.062527220, Longitude=-91.64848880). The Old River Outflow Channel diverts about a third of the streamflow from the mainstem of the Mississippi River, thus to capture the total amount of water and nutrients to the Gulf of Mexico, streamflow flowing through the Old River Control structure and below were summed. Total drainage area of the MRO is 2,914,514 km^2 and 99.08% of the basin is within the US, so that total MRO drainage area in the United States is 2,887,854 km^2 and includes the Arkansas (excluding Red River Basin), Ohio, Tennessee, Missouri, and Upper Mississippi River basins. The nutrient balances reflect the inputs and outputs from this area (Figure S2).

Figure S1. This study reports the water quality load trends for one site on the Mississippi River, which we refer to as the Mississippi River Outflow (MRO). Water quality data to estimate loads were from the Mississippi River near St. Francisville. The discharge data were from the Mississippi River at Tarbert Landing and the Old River Outflow Channel (both measured by the U.S. Army Corps of Engineers). Original figure in Murphy et al. (2013).

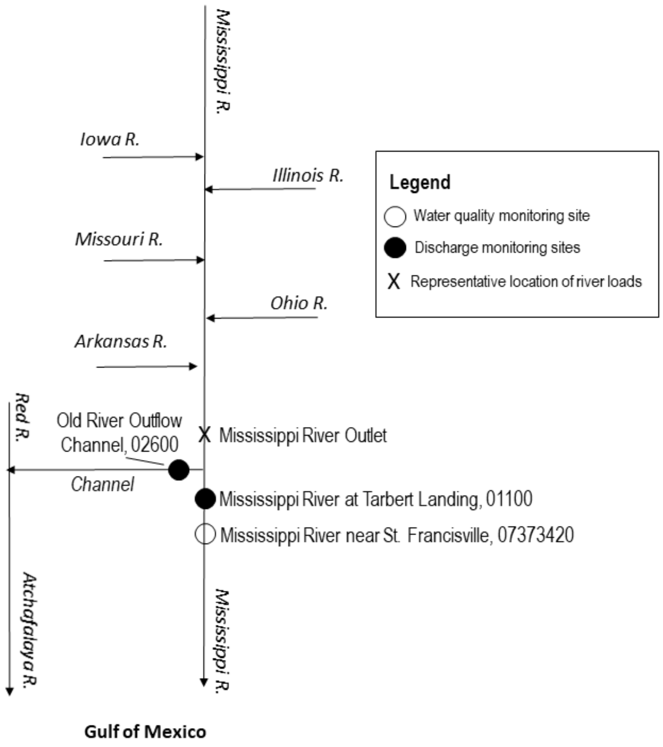

Section 2 – Methods

River Loads

The water quality data used in the river nutrient and sediment load trend analysis are shown in Table S1. Sample collection, analysis, and data preparation procedures are described at the US Geological Survey (USGS) National Water Quality Network website (Aulenbach et al. 2007, US Geological Survey 2019) and the TP and NH3 concentration data reflect additional re-censoring identified in USGS Technical Memoranda (Aulenbach et al. 2007, Oelsner et al. 2017). The period of record for all constituents was water years 1975 to 2017, with the exception of Suspended Sediment, which was from 1978 to 2017. Total Nitrogen is reported as the sum of Kjeldahl nitrogen (00625), which is ammonia plus organic nitrogen, and nitrate plus nitrite (00631). Suspended sediment describes the concentration of solid-phase material (sand, silt, clay) suspended in a water-sediment mixture.

Table S1. Information about the water quality data used to in the WRTDS river nutrient and sediment load and trend analysis. No censored values were used in the analysis.

Constituent	Constituent Acronym	USGS Parameter Code	Minimum Concentration Average mg L^{-1}	Maximum Concentration Average mg L^{-1}	Number of Concentration Samples
Total Phosphorus	TP	00665	0.05	0.8	547
Orthophosphate	OP	00671	0.01	0.19	423
Total Nitrogen	TN	00631 plus 00625	0.8	4.4	491
Nitrate plus Nitrite	NO23	00631	0.23	3.2	558
Ammonium	NH3	00608	0.005	0.32	486
Suspended Sediment	SS	80154	19	1020	450

Discrete water quality data, daily streamflow records, and the Weighted Regression on Time, Discharge, and Seasons (WRTDS) model (Hirsch et al. 2010) were used to estimate river loads. One of the key benefits of using the WRTDS model for long-term water quality trend analysis is that it allows for flexibility in the relationship between concentration and flow over time, which has been shown to improve load estimation accuracy and lower bias (Hirsch et al. 2010, Lee et al. 2017). Input data to the WRTDS model were screened to ensure that an adequate number of concentration samples were available on a seasonal and annual basis and to assure sufficient sampling occurred across a range of hydrologic conditions, including high flow events (Oelsner

et al. 2017). The WRTDS model was run at a daily time step, and the loads were aggregated to annual means. Model performance evaluations, including a visual assessment of model fit and the detection of bias in residuals were completed, and models showing problems in either were excluded from the final set of results.

In this study, the trends in nutrient loads were determined using an enhancement of WRTDS. In the original implementation of WRTDS, flow normalization was based on the assumption that the flow regime for the trend period of record is the same; meaning that the probability distribution of daily annual flows was stationary from the beginning to the end of the period of record (Murphy and Sprague 2019). Essentially, the flow normalization removes the effects of random and systematic changes in streamflow on nutrient loads. In the enhancement, the overall trend in water quality is the sum of two components. In the first component of the enhancement, the probability distribution of discharge is stationary. The trend results therefore show the effect of changes in water quality apart from changes in discharge and serve as an indicator for the effects of human activities and decisions, including nutrient management, on water quality conditions over time. The second component of the trend in water quality is the results of changes in the probability distribution of discharge over the period of record, and account for the amount of change in the overall trend that can be attributed to changes in the streamflow regime. We found that the first component of the water quality trend accounted for 98% of the water quality trends for all six constituents, indicating that the trends were not attributable to changes in the streamflow regime, but were instead attributable to changes in management. Therefore, the trend attribution analysis was focused on linking the water quality trends with nutrient balances. Confidence intervals on trends in FN loads were estimated using a block bootstrapping approach (Hirsch et al. 2015).

Nutrient Balances

Description of Nutrient Balances

Nutrient balances were calculated as the difference between inputs and outputs. The N and P balances were calculated as:

N balance (kg km^{-2}) = $(N_{fertilizer} + N_{manure} + N_{wastewater} + N_{fixation} + N_{atmosphericdeposition}) - (N_{cropuptake} + N_{emissions})$ (Equation 1)

P balance (kg km^{-2}) = $(P_{fertilizer} + P_{manure} + P_{wastewater} + P_{weatheredrock}) - (P_{cropuptake})$ (Equation 2)

The N balance *inputs* include N retained in fertilizer, N retained in manure, N in waste water treatment facility effluent, N from N_2O fixation, and N deposition and *outputs* include N harvested and removed in crops, hay, and pasture and N lost to the atmosphere. The P balance *inputs* include P retained in fertilizer, manure, waste-water treatment facility effluent, and weathering and *outputs* include P harvested and removed in crops, hay, and pasture. Nutrient balances were estimated from 1950 to 2017 for the MRO (Figure S2).

Figure S2. Map of drainage area used in nutrient balance estimates. Drainage area used in this study includes areas upstream from the MRO.

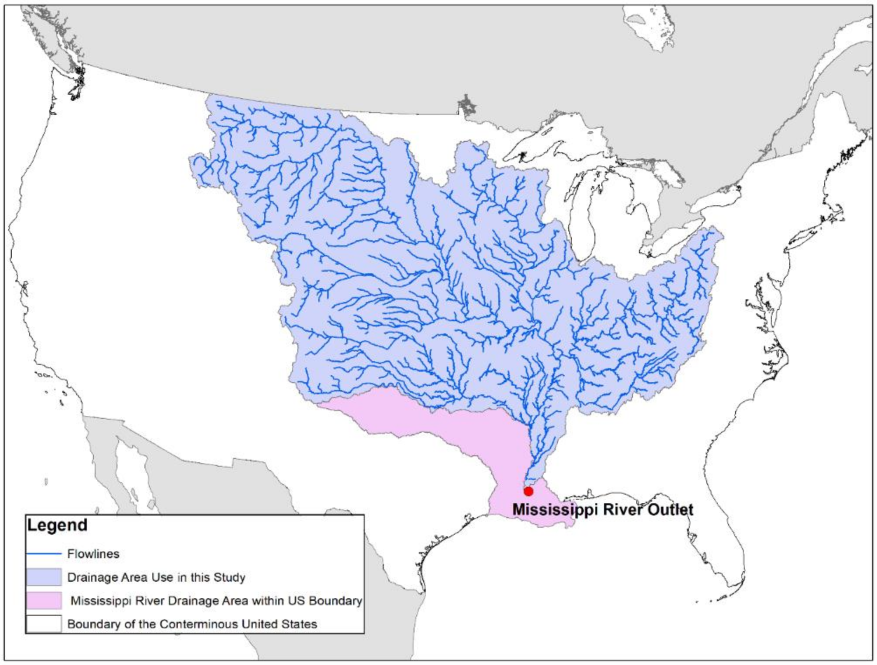

Table S2. Sources, period of record, and periodicity of input and output datasets used in estimating N and P balances from 1950 to 2017 for the MRO.

Dataset	Source Data	Start Date	End Date	Periodicity	Missing Data Fill Method
Fertilizer	(Falcone 2021b)	1950	2017	Annual, missing 2013, 2014, 2015 & 2016	Interpolation
Manure	(Falcone 2021b)	1950	2017	Every 5 years	Interpolation
N_2O Fixation	Crop Yield	1950	2017	Every 5 Years	Interpolation
Atmospheric Deposition	(US Environmental Protection Agency 2020)	1950	2017	Annual	Not applicable
Waste Water Treatment Effluent	(Falcone 2017)	1978	2012	Biennially from 1978 to 1992 then every four years from 1992 to 2012	Imputation
Phosphorus Weathering	Terziotti 2019	Static	Static	Static Variable	Static
Crop Yield	(Falcone 2020)	1950	2017	Every 5 years	Interpolation
N Emissions	Fertilizer and manure (this study)	1950	2017	1950 to 2017	Used Interploated Fert ilizer and Manure (this study)
River Load	This study	1975	2017	Annual	Imputation

Nutrient Balances Input Datasets

Fertilizer. Our fertilizer dataset for 1950 to 2017 integrated fertilizer data from 3 different time periods, 1950 to 1985, 1986 to 2012, and 2013 to 2017. For the period 1950 to 1985, state-level fertilizer sales data were obtained through state-level Department of Agriculture fertilizer use, reported as total mass (Alexander and Smith 1990). These values were then allocated to counties, by assuming that the county level fertilizer use was directly proportional to the amount of fertilized acreage present within the counties. Fertilized acreage came from the US Department of Agriculture Census of Agriculture (CoA) (Alexander and Smith 1990). For 1950-1985 only total fertilizer numbers were available, however non-farm fertilizer was estimated from the proportion of farm and non-farm fertilizer data for 1987-2012. Based on the data from 1987 – 2017, 98.6% of total P and total N were estimated for farm and 1.4% for non-farm fertilizer for the years before 1987.

For the period 1987 to 2012, state level fertilizer sales data were from the Association of American Plant Food Control Officials (AAPFCO). These values were then allocated to the county-level using fertilizer expenditure data from the Census of Agriculture as county weights (Gronberg and Spahr 2012, Brakebill and Gronberg 2017). For time period 1987 to 2012, non-farm fertilizer was estimated using fertilizer sales data above, and then allocated spatially based on urban land cover in the county (Gronberg and Spahr 2012).

For the period 2013 to 2017, farm fertilizer for 2017 was estimated using a regression of county-level Census of Agriculture crop and fertilizer expenditure variables against nutrient values for the three censuses preceding it (2002, 2007, and 2012). For 2017, values of non-farm nutrients from fertilizer were estimated based on population, as described in (Falcone 2021a).

Once all county-level data were assembled (Falcone 2021b) annual values for the MRO were calculated by intersecting the watershed boundary and counties and extracting annual mean value for farm and non-farm fertilizer for each year. Estimates of N and P from farm and non-farm fertilizer for years with missing values (2013 to 2016) were interpolated using the *na.approx* function in R (R Studio, 1.2.5033).

The estimated annual farm and non-farm N and P from fertilizer for the time period 1950 to 2017 used in the nutrient balances are shown in Figure S3.

Figure S3. Nutrient masses per area per year for A) farm-fertilizer nitrogen B) non-farm fertilizer nitrogen, C) farm-fertilizer phosphorus, and D) non-farm fertilizer phosphorus (kg km^{-2}) from 1950 to 2017 for the MRO. Interpolated values are shown in grey for years 2013 to 2016.

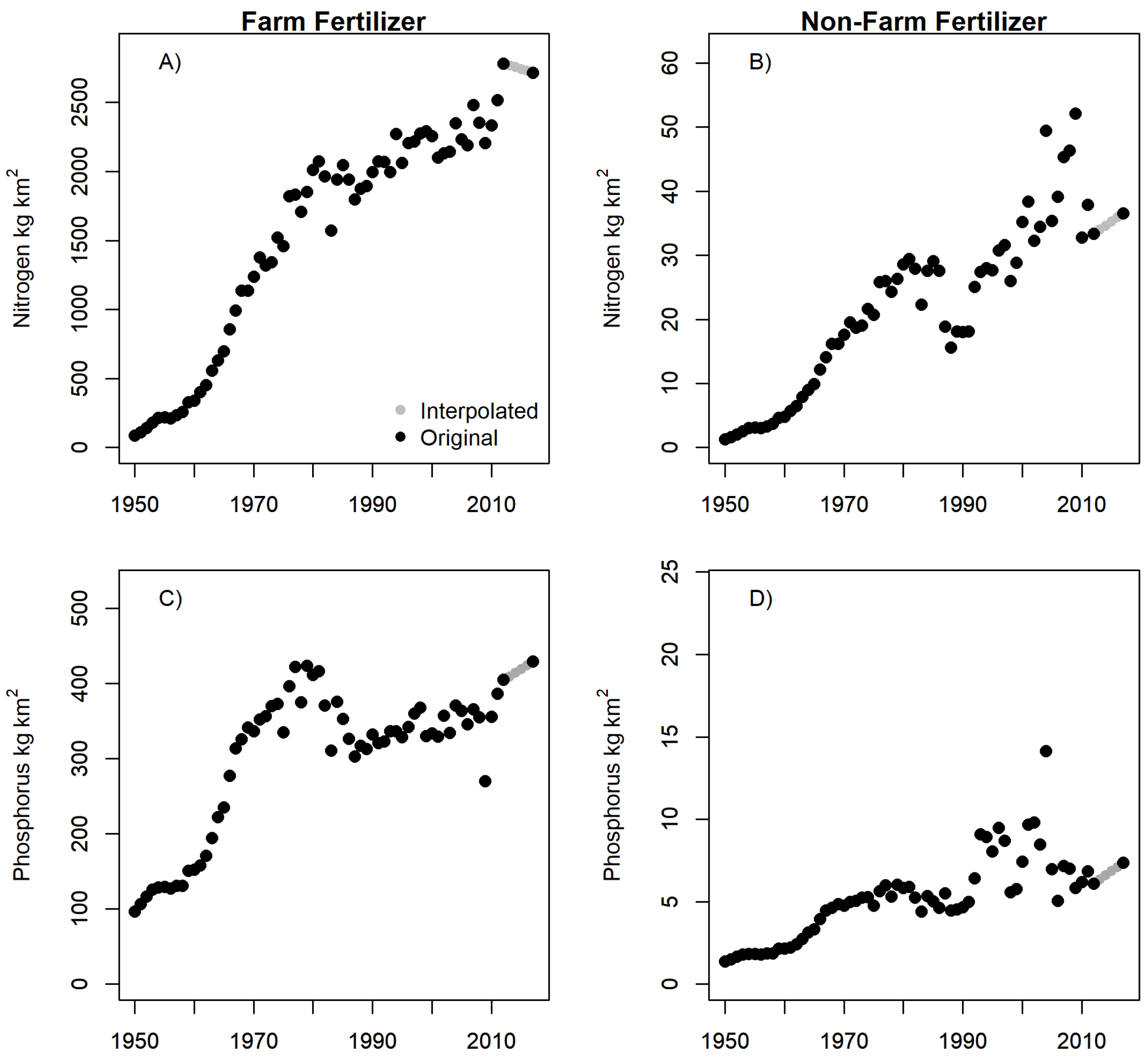

Manure. Estimates of total N and P in manure were based on animal life span (days), animal population inventories (animals), and estimates of manure nutrient content (kg animal^{-1} day^{-1}) as in (Mueller and Gronberg 2013, Gronberg and Arnold 2017). Livestock-specific manure N and P content incorporated changes in animal weight over time (Falcone 2021a). The number of animals per county were taken from Census of Agriculture data for census years between 1950 and 2017 and changes in animal weights were obtained from the USDA Economic Research Service, as described in Falcone (2021a). Estimates of animal counts were made for counties which had data withheld for privacy purposes by the CoA. Once all county-level data were assembled (Falcone 2021b), annual values for the MRO were calculated by intersecting the watershed boundary and counties and extracting annual mean value for manure for each year (Figure S4). The Census of Agriculture animal and crop production data were available every four to five years for the time period from 1950 to 2017, therefore manure N and P estimates were only available every four to five years. However, annual estimates were needed for the subsequent nutrient balance computations. Estimates of manure N and P for years with missing values were interpolated using the *na.approx* function in R (R Studio, 1.2.5033).

Figure S4. Nutrient masses per area per year for A) manure N and B) manure P for the MRO from 1950 to 2017.

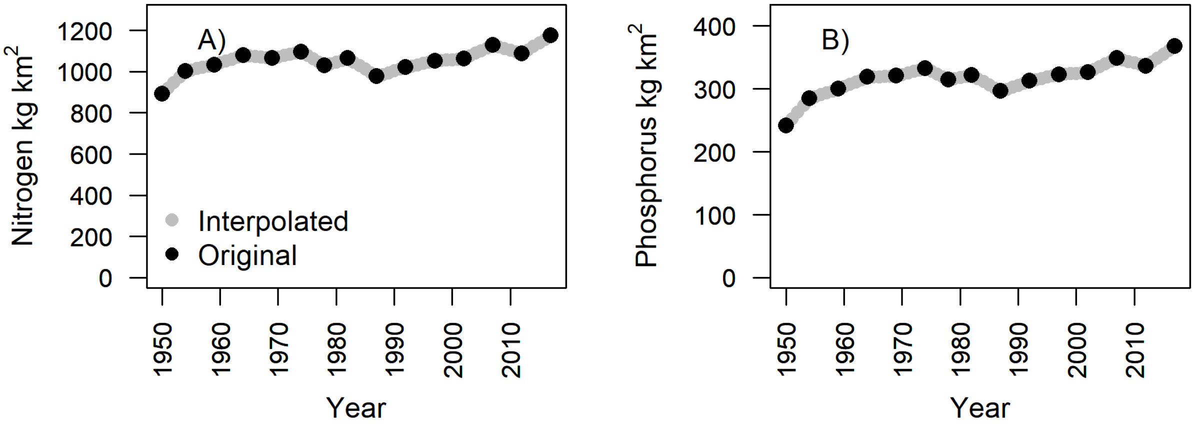

Waste Water The EPA Clean Water Needs Survey (CWNS) data provided waste-water treatment facility (WWTF) nutrient inputs to rivers based on annual outflow (water discharge/day) and water treatment level categories. Treatment level categories are primary, secondary, and advanced. Primary treatment uses physical means to remove solids from raw sewage, and secondary and advanced use biological methods to remove nutrients from wastewaters. Each treatment category had an average total nitrogen and phosphorus concentration drawn from the literature. Because the CWNS data used here somewhat under-represent total WWTF sites (most major dischargers are included but some minor ones are not), the effluent estimates given here similarly under-represent the total effluent load (Ivahnenko 2017). Biennial Total N and Total P WWTF effluent estimates (kg km^{-2}) were available for the time period 1978 to 2012. WWTF effluence data were available biennially from 1978 to 1992 then every four years from 1992 to 2012. Missing values between 1950 to 2017 were estimated using the “estim_ncpPCA” and “imputePCA” functions in the missMDA package (version 1.18) (Husson and Josse 2015).

Figure S5. Nutrient masses per area per year for A) nitrogen in Waste-Water Treatment Facility (WWTF) effluent and B) phosphorus in WWTF effluent for the MRO from 1950 to 2017.

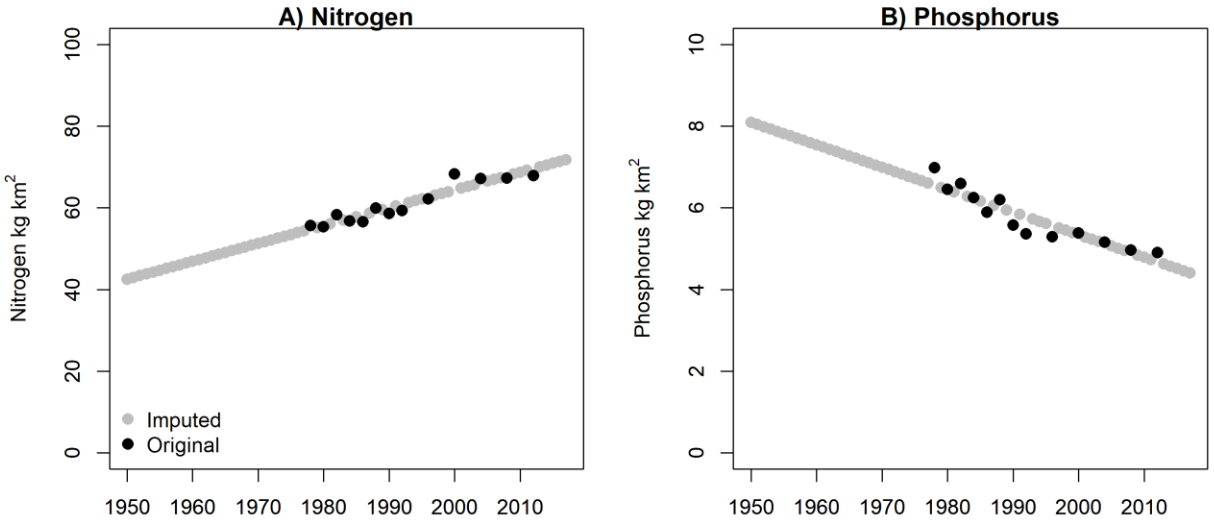

Nitrogen-fixation Dinitrogen gas is the most abundant form of N in the biosphere, but it is unusable by most organisms. Biological N_2 fixation is the dominant process by which N_2 is transformed into organic N (Robertson and Groffman 2007). We compared methods for

estimating N-fixation for four different crops: soybean, alfalfa hay, and non-alfalfa hay, and pasture. Alfalfa hay is comprised of the leguminous species *Medicago,* and non-alfalfa hay contains some leguminous species, like vetch (*Vicia*) and clover (*Trifolium*), but also contains grass species, which do not have an N-fixing capacity.

For the first method, referred to as Sabo 2019, N_2 fixation rates were based on plant yield (2019). This method is based on an approach established by the International Plant Nutrient Institute (IPNI), which estimated N-fixation rates as equal to nitrogen uptake and removal in crop harvest (International Plant Nutrition Institute 2012). The next two methods used a fixed N_2 fixation rate multiplied by crop yield or area. These two methods are referred to as Sprague 2012 and Swaney 2018. N-fixation rates of 0.91 kg bushel^{-1} from Sprague (2012) and 1.78 kg bushel^{-1} from Swaney (2018) were multiplied by crop yield (bushel km^{-2}) to estimate N-fixation rates for soybean. Swaney used N-fixation rates of 28.12 kg dry ton^{-1} for alfalfa hay (Medicago) and 3.08 kg dry ton^{-1} for non-alfalfa hay, while Sprague used area-based coefficients for alfalfa hay (218 kg hectare^{-1}) and non-alfalfa hay (116 kg hectare^{-1}). N-fixation rates in pasture were not calculated in the Sprague 2012 method. The fourth method, which was area based, is referred to as Boyer 2002. The N-fixation rate for soybean was 9600 kg km^{-2}, alfalfa hay was 22400 kg km^{-2}, non-alfalfa-hay was 11,700 kg km^{-2}, and pasture was 1500 kg km^{-2} (Boyer et al. 2002).

These methods relied on either crop yield or crop area data that was only available every 5 years. Estimates of crop yield and area for years with missing values were interpolated for each crop type using the *na.approx* function in R (R Studio, 1.2.5033) (Outputs Section; Figure S9). Figures S6 and S7 show time series of N_2 fixation rates based on these interpolated crop yields and areas. The Sprague 2012 and Boyer 2002 approach had lower N-fixation rates for soybean and higher rates for both types of alfalfa than the Sabo 2019 and Swaney 2018 method (Figure S6). The Sabo 2019 and Swaney 2018 estimates are similar across the entire period of record indicating a convergence on N-fixation rates using yield-based approaches (Figure S7). The Sabo 2019 approach was chosen the preferred method to use in the N balances.

Figure S6. Nutrient masses per area per year for A) soybean N-fixation, B) alfalfa hay N-fixation, C) non-alfalfa hay N-fixation, and D) pasture N-fixation for the MRO from 1950 to 2017. Original and interpolated crop yield data are shown in Figure S9. For Panel C, the Sprague 2012 results are the same as the Boyer 2002 results. For panel D, there were no Sprague 2012 results, and the Boyer 2002 results were the same as Swaney 2018.

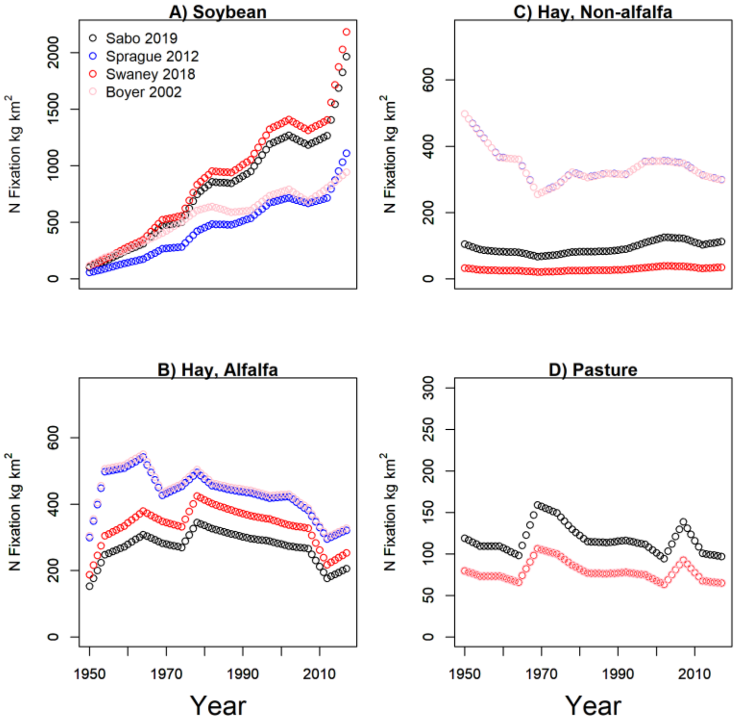

Figure S7. Estimated total N-fixation rates (sum from soybean, alfalfa hay, non-alfalfa hay, and pasture) for the Mississippi River Basin. Four methods are shown: IPNI, Sprague et al. 2012, Swaney et al. 2018, and Boyer 2002. Original and interpolated crop yield data are shown in Figure S9.

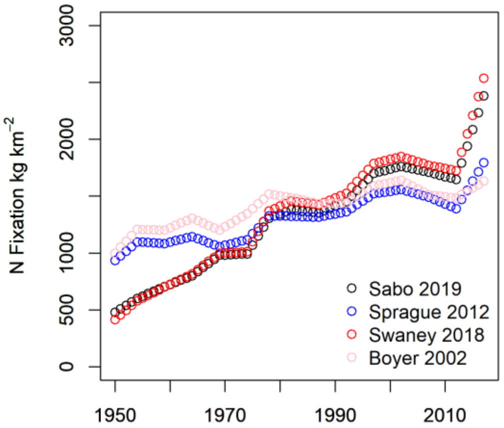

Atmospheric Deposition. N atmospheric deposition estimates were derived from the EPA Critical Loads (CL) estimates website (US Environmental Protection Agency 2020). The Coupled Modelled Intercomparison Project Phase 5 (CMIP5) 1850 – 2000 estimates were used for 1950 to 2000 in our study. CMIP5 focused on improving representation of climate and consists of a set of climate model experiments. The historical (1850-2000) wet and dry nitrogen (N) and sulfur (S) deposition estimates included in the CL Mapper Tool represent gridded emissions of reactive gases and aerosols for use in chemistry model simulations needed by climate models for the CMIP5 in support of the Intergovernmental Panel on Climate Change (IPCC) Fifth Assessment report (AR5). The emissions were developed using RETRO (1960–2000) and EDGAR-HYDE (1890–1990) emissions and version 3.5 of the global Community Atmosphere Model (CAM) (Lamarque et al. 2010). The Total Deposition (TDEP) estimates were used for the 2001 to 2017 period of record in our study. TDEP deposition estimates are developed from an approach which combines measured (NADP and Clean Air Status and Trends Network – CASTNET) and modeled (Community Multiscale Air Quality – CMAQ) values, with the measured values being given greater weight at monitoring station locations, and modeled data providing values for locations in between the stations and for chemical species not measured by the monitoring networks. N deposition data included reduced (NH_3), oxidized [NO_x (NO, NO_2, NO_3), N_2O_5, HNO_3, PAN (peroxyacetylnitrate), aerosol nitrates, and other organic nitrates]

and total N (Figure S4). Atmospheric ammonia and organic nitrogen largely are derived from volatilization of animal waste and fertilizer. Because these emissions may redeposit during the same year in close proximity to the emission source (Prospero et al., 1996), deposition of ammonia and organic nitrogen were assumed to be recycled from other inputs in the same region and were not included as inputs in this study (Jordan and Weller 1996, McIsaac et al. 2002, Sprague and Gronberg 2012).

Figure S8. Annual N Wet and Dry Atmospheric Deposition rates for the Mississippi River Basin 1950 – 2017. The only atmospheric N species included in the nitrogen balances was oxidized-N. Total N and reduced N are shown for comparison.

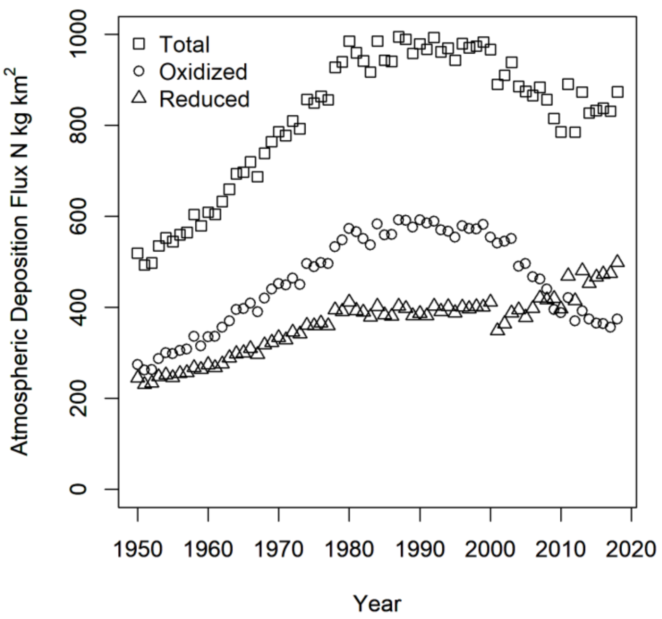

P from weathering. The amount of P from weathering was estimated using information from two datasets. The first dataset provided estimates of the abundance and spatial distribution of chemical elements and minerals in soils of the conterminous United States and represented a baseline for soil geochemistry and mineralogy against which future changes may be recognized and quantified (Smith et al. 2014). The dataset was created through the analysis of soil samples collected at depth of 0 to 100 cm from 4,857 sites distributed across the CONUS. Field sampling

was completed in 2010, and the soil samples were translated into a continuous map of available P in the A horizon (Terziotti 2019). From the map, we selected 2,272 points in "natural" settings; natural was defined as more than 90% non-agricultural and non-urban 2008 land use in a 1-kilometer buffer around the point. We used the points from natural settings because we wanted to exclude the potential of legacy P concentrations in the soil from anthropogenic influences, including past agricultural activities. From those 2,272 data point in natural settings, a national surface of “natural P” was made using Inverse Distance Weighting based on the methods of Terziotti (2019). From this we estimated that the average soil concentration for the MRO was 504 ppm. We multiplied the 504 ppm soil concentration by the average bulk density for the MRO of 1.42 (g cm^{-3}) (Falcone 2017) to derive a soil P stock of 716 kg km^{-2}. Robertson et al (2019) estimated that about 1% of the background material is a source of P to rivers and streams, and 1% of 716 is equal to 7.16 kg km^{-2} yr^{-1}. We verified this value of P weathering rates by estimating average P weathering rates from a different data source; sedimentary rocks from a global geodatabase that describes weathering and P release (Hartmann et al. 2014). We multiplied P weathering rates from a range of lithological classes by the number of pixels represented by each class within the MRB (see Table S3). Using this approach, the average weathering rate across the MRO was 7.16 kg km^{-2} yr^{-1}, equal rate calculated using the first approach. Our average weathering rate corresponds to published literature estimate of background P sources contributing to rivers and streams of 10 kg km^{-2} yr^{-1} (Withers and Jarvie 2008). We used this average weathering rates as a P input to the P balances for years 1950 to 2017, recognizing that this is not a comprehensive representation of P weathering rates; there can be hotspots of weathering and release of P from soils, and weathering rates respond to variability in temperature and precipitation.

Table S3. P weathering in the Mississippi River Basin based on lithology type (Hartmann et al. 2014) and number of pixels associated with each lithology type. The average total P (9164 kg P km^{-2} yr^{-1}) divided by the number of ½ degree pixels (1280) produced an average weathering rate of 7.16 kg P km^{-2} yr^{-1} for the MRO. Data were derived from the supplemental map: https://doi.pangaea.de/10.1594/PANGAEA.788537

Lithology Type	P weathering rate (kg P km^{-2} yr^{-1})	Number of pixels	kg P km^{-2} yr^{-1} from Each Type
Unconsolidated Sediments (SU)	6.28	295	1852.6
Basic Volcanic Rocks (VB)	29.77	5	148.85
Siliciclastic Sedimentary Rocks (SS)	4.48	573	2567.04
Basin Plutonic Rocks (PB)	37.17	0	0
Mixed Sedimentary (SM)	6.56	111	728.16
Carbonate Sedimentary Rocks (SC)	14.46	214	3094.44
Acid Volcanic Rocks	3.92	5	19.6
Metamorphics (MT)	4.41	33	145.53
Acid Plutonic Rocks (PA)	6.63	22	145.86
Intermediate volcanic rocks (VI)	37.54	12	450.48
Water Bodies	0	8	0
Pyroclastics (PY)	84.71	0	0
Intermediate Plutonic Rocks (PI)	10.92	1	10.92
Evaporites (EV)	1.49	1	1.49
No Data (ND)	0	0	0
Ice and Glaciers (IG)	0	0	0
Totals		1280	9164.97
**Mean (Total Weathering Divided by Number of Pixels)**			**7.16**

Nutrient Balances – Output Datasets

Crop Nutrient Uptake and Harvest Removal. Crop Uptake and Harvest Removal was estimated as: Watershed Crop N or P Uptake and Harvest Removal =∑ (Crop Nutrient Content$_i$ x Crop Yield$_{ij}$), where *i* stands for an individual crop, and *j* stand for year. *Crop Nutrient Content* (kg N or P crop unit^{-1}) values (Russell et al. 2008, Sprague and Gronberg 2012, Swaney et al. 2018) were constant throughout the study period (1950-2017). *Crop yield* values were based on Census of Agriculture (CoA) data (LaMotte 2015) and varied by year. CoA yield data were available on average every 5 years between 1950 to 2017: 1954, 1959, 1964, 1969, 1974, 1978, 1982, 1987, 1992, 1997, 2002, 2007, 2012, 2017. Estimates of cropland harvested crops for years with missing values were interpolated using the *na.approx* function in R (R Studio, 1.2.5033). The yields and areas used in the crop uptake and harvest removal and the N2 fixation results, including the interpolated values, are summarized in Figure S9. Estimates of N and P take up by crops and removed in harvest are shown in Figure S10.

The total area covered by the pasture 12 crops and pasture included in this study (Table S4) represents 94% of all harvested cropland area in the MRO.

Table S4. Conversion factors used to estimate crop N and P uptake and harvest removal for 12 crops and pasture.

Crop	Crop Unit	Crop Nutrient Content, kg N per crop unit	Crop Nutrient Content, kg P per crop unit	Source of conversion factors
Barley	bushels	0.41	0.08	Sprague
Corn grain	bushels	0.36	0.07	Sprague
Corn silage	green tons	3.22	0.48	Sprague
Cotton	bales	6.86	1.29	N, Swaney; P, Russell
Hay - Alfalfa	dry tons	22.86	2.14	Sprague
Hay – Non-alfalfa	dry tons	9.85	5.30	Sprague
Oats	bushels	0.27	0.05	Sprague
Rice	hundredweight	0.57	0.13	Sprague
Sorghum grain	bushels	0.44	0.08	Sprague
Sorghum silage	green tons	6.70	1.11	Sprague
Soybean	bushels	1.61	0.16	Sprague
Wheat	bushels	0.56	0.10	Sprague
Pasture	acres	9.07	5.00	Sprague

Figure S9. Original and interpolated crop yield and area estimates from 1950 to 2017 for the Mississippi River Basin. Yields for 12 crops and pasture are shown in panels A - M, and areas for 4 crops are shown in panels N - Q.

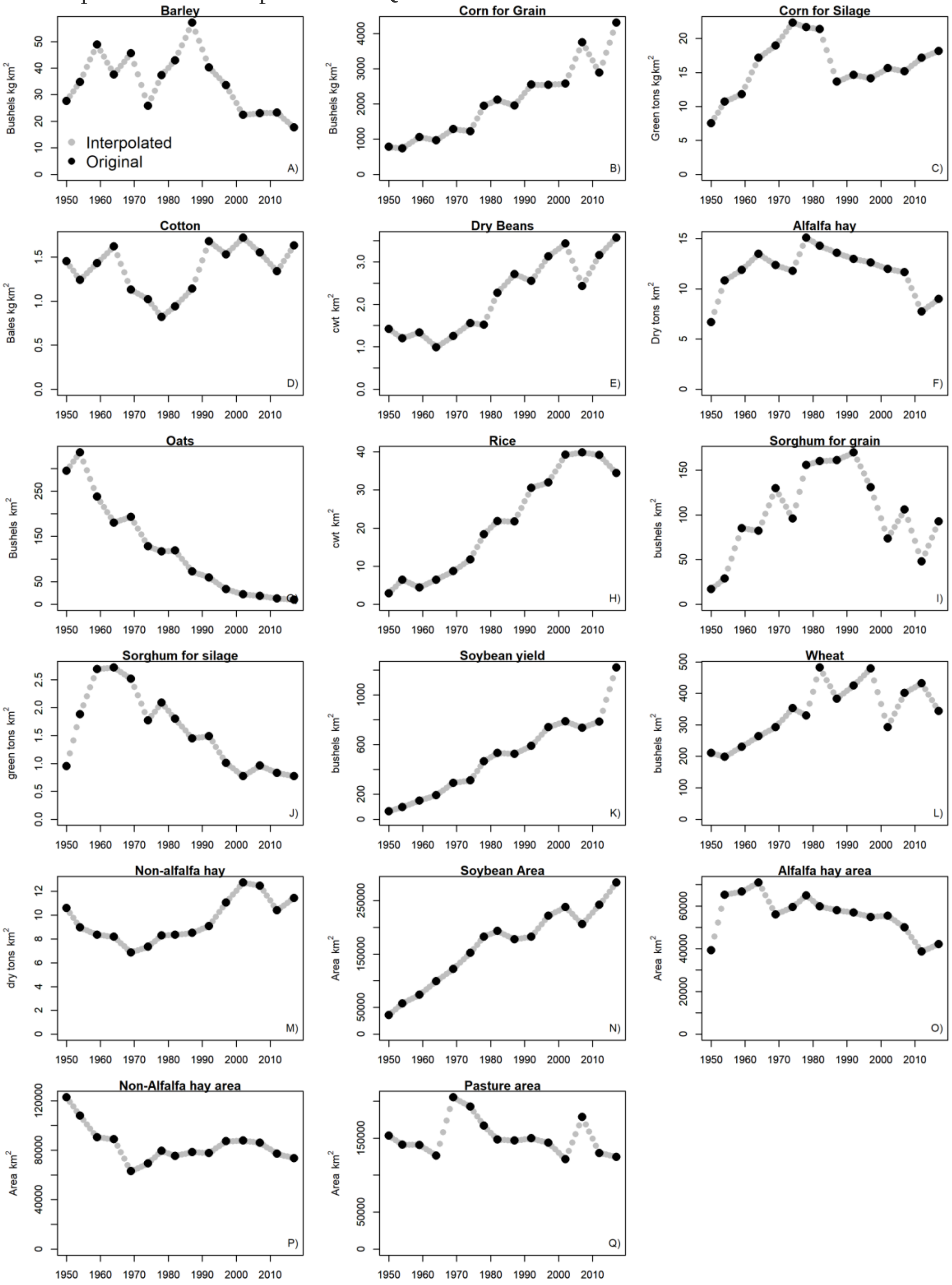

Figure S10. Nutrient masses per area per year for A) N uptake and removal in harvest, and B) P uptake and removal in harvest for 12 crops and pasture in the MRO for the time period 1950 to 2017.

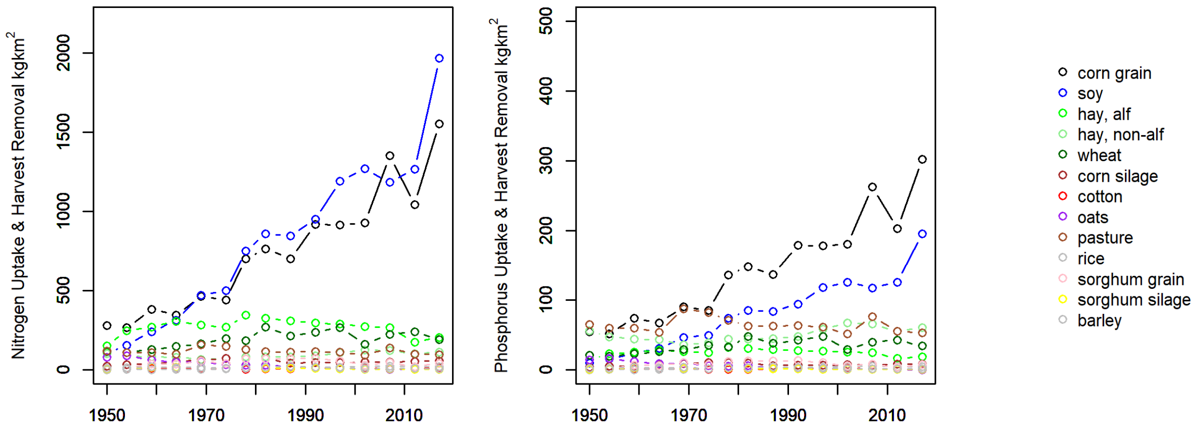

N Emissions. Two gaseous denitrification by-products were considered N outputs, N_2O and N_2 emissions from fertilizer, manure, and soils using emission factors from De Klein et al. (2006). Emissions of N_2O was the sum of two components (Sabo et al. 2019). The first component, N emissions from fertilizer and manure uses emission factor one (EF1), which assumes that 1% of fertilizer and manure was emitted as N_2O. Therefore, we multiplied farm fertilizer, non-farm fertilizer, and manure by 0.01 and then summed them together, giving us emissions from EF1. The second emission factor, EF2, N emissions from soils and pastureland uses emission factor 2, which assumes that 200 kg km^{-2} of N_2O is emitted from organic soils and pastureland. To estimate total N_2O emissions, we summed emissions from EF1 and EF2. To estimate N_2 emissions, we assumed that 17% of the total N_2O emissions were emitted to the air as N_2 gas (Sabo et al. 2019). We also include N emissions from river surfaces. Our estimate was static over time and was based on Spatially Referenced Regression on Watershed Attributes (SPARROW) model results for the time period of 2000 to 2014, which estimated N sources to rivers and streams were 650 kg km^{-2} yr^{-1}, and 19% of these N sources were lost annual from the river surfaces to the atmospheric (Robertson and Saad 2019).

Trend Attribution

Regression Model

This section describes the correlation analysis between time-lagged nutrient balances and river loads, a process that we used to determine the most relevant time-lagged nutrient balances to pass on to the model selection process, which was key component of the development of our regression model. Previous work has used time lags of nutrient balances and river discharge to explain 95% of the variability river nitrate loads from 1960 to 1998 (McIsaac et al. 2002). This study serves as a continuation and expansion of that work investigating impacts of legacy nutrients on river loads, as we provided an updated regression model using current (contemporary) and time-lagged (legacy) nutrient balances as explanatory variables for both TN and TP. Legacy nutrients were represented in the model with N and P balances that were sequentially shifted in one-year increments up to a lag of 25 years. For example, for a ten-year lag, the 1976 river load was matched with balance estimate for 1966.

Figure S11. Lag correlation plot of flow-normalized river loads with nutrient balances, 1975 to 2017. Pearson r values above 0.3 or below -0.3 (indicated by the dashed line) indicate significant correlations (p-values <0.05).

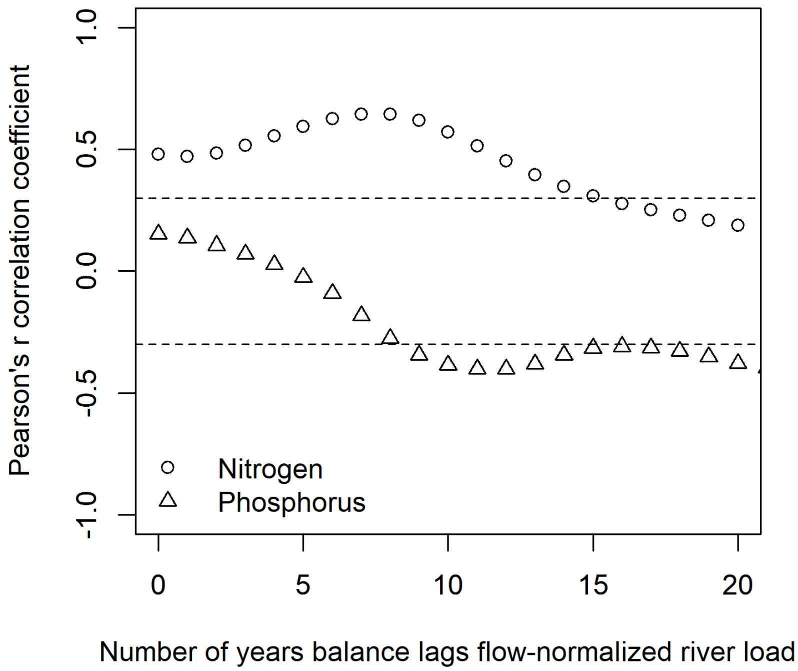

Table S5. Pearson's R Correlation results. N and P nutrient balances were lagged 0 to 25 years. R-squared and p-values for the correlation of each time lag with river TN and TP loads are reported.

Time-lag for nutrient balances	Correlation with TN River Loads Pearson's R Correlation Coefficient	p-value	Correlation with TP River Loads Pearson's R Correlation Coefficient	p-value
0	0.4789	0.0012	0.1536	0.3255
1	0.4697	0.0015	0.1368	0.3818
2	0.4849	0.0010	0.1063	0.4975
3	0.5170	0.0004	0.0714	0.6491
4	0.5541	0.0001	0.0276	0.8604
5	0.5927	0.0000	-0.0246	0.8757
6	0.6261	0.0000	-0.0914	0.5598
7	0.6433	0.0000	-0.1828	0.2408
8	0.6430	0.0000	-0.2751	0.0742
9	0.6182	0.0000	-0.3432	0.0242
10	0.5702	0.0001	-0.3840	0.0110
11	0.5128	0.0004	-0.4012	0.0077
12	0.4521	0.0023	-0.4002	0.0078
13	0.3950	0.0088	-0.3804	0.0118
14	0.3477	0.0223	-0.3445	0.0237
15	0.3096	0.0434	-0.3151	0.0396
16	0.2770	0.0721	-0.3082	0.0443
17	0.2507	0.1048	-0.3136	0.0406
18	0.2286	0.1403	-0.3281	0.0317
19	0.2080	0.1808	-0.3512	0.0209
20	0.1884	0.2262	-0.3774	0.0126
21	0.1732	0.2666	-0.3961	0.0086
22	0.1641	0.2930	-0.3906	0.0096
23	0.1599	0.3057	-0.3542	0.0198
24	0.1600	0.3054	-0.2946	0.0552
25	0.2723	0.0773	-0.2180	0.1603

Counterfactual Analysis

The third component of our trend attribution was an impact evaluation. Specifically, we wanted to determine if nutrient management, expressed in this study as current and time-lagged N and P balances, or background processes for which we don't have data, which were expressed in this study in the year term, affected progress toward meeting the specific goal reducing nutrient delivery to the Gulf of Mexico (Ferraro 2009). The impact analysis was framed using a counterfactual approach. Counterfactuals formally compare what actually happened to what would have happened, and we examined what river loads would have looked like without the observed patterns in nutrient balances or background processes. For Counterfactual A, the nutrient management terms, including the current year nutrient balances, as well as any lagged balance terms, were held constant after 1975. The objective of holding the balance term constant was to investigate downstream water quality in the absence of any variability in nutrient management after 1975. Holding the balance term constant may affect the magnitude of both current and legacy sources, depending on the variables represented in the N and P regression models. For the Counterfactual B, we held the independent Year terms of the N and P regression models constant. The current and time-lagged nutrient balance terms in the input datasets for counterfactual B were not altered; they were the same as in the regression model. Here we show the N and P input dataset for Counterfactuals A and B (Tables S6 – S9). We also provide a plot of the 2-year time lagged N balance and the 4-year time lagged P balances the N and P input dataset for Counterfactual A as compared to the original data to demonstrate the differences in Counterfactual A input data, as compared to the original data used in the regression model.

Table S6. Input dataset for Nitrogen Counterfactual A. Columns represent significant variables for the N regression model, Year, Year2, and the 2, 4,9 and 11-year time-lagged nitrogen balances. Counterfactual A held the 4 balance terms constant at 1975 levels after the appropriate lag of 2,4,9, and 11 years.

YEAR	YEAR	$YEAR^2$	N_BALANCE_KGSQKM_2LAGYR	N_BALANCE_KGSQKM_4LAGYR	N_BALANCE_KGSQKM_9LAGYR	N_BALANCE_KGSQKM_11LAGYR
1975	-21	441	1405.461	1416.574	945.8797	780.4774
1976	-20	400	1642.159	1384.349	1024.148	809.2727
1977	-19	361	1487.939	1405.461	1165.054	945.8797
1978	-18	324	1487.939	1642.159	1150.443	1024.148
1979	-17	289	1487.939	1487.939	1273.053	1165.054
1980	-16	256	1487.939	1487.939	1416.574	1150.443
1981	-15	225	1487.939	1487.939	1384.349	1273.053
1982	-14	196	1487.939	1487.939	1405.461	1416.574
1983	-13	169	1487.939	1487.939	1642.159	1384.349
1984	-12	144	1487.939	1487.939	1487.939	1405.461
1985	-11	121	1487.939	1487.939	1487.939	1642.159
1986	-10	100	1487.939	1487.939	1487.939	1487.939
1987	-9	81	1487.939	1487.939	1487.939	1487.939
1988	-8	64	1487.939	1487.939	1487.939	1487.939
1989	-7	49	1487.939	1487.939	1487.939	1487.939
1990	-6	36	1487.939	1487.939	1487.939	1487.939
1991	-5	25	1487.939	1487.939	1487.939	1487.939
1992	-4	16	1487.939	1487.939	1487.939	1487.939
1993	-3	9	1487.939	1487.939	1487.939	1487.939
1994	-2	4	1487.939	1487.939	1487.939	1487.939
1995	-1	1	1487.939	1487.939	1487.939	1487.939
1996	0	0	1487.939	1487.939	1487.939	1487.939
1997	1	1	1487.939	1487.939	1487.939	1487.939
1998	2	4	1487.939	1487.939	1487.939	1487.939
1999	3	9	1487.939	1487.939	1487.939	1487.939
2000	4	16	1487.939	1487.939	1487.939	1487.939
2001	5	25	1487.939	1487.939	1487.939	1487.939
2002	6	36	1487.939	1487.939	1487.939	1487.939
2003	7	49	1487.939	1487.939	1487.939	1487.939
2004	8	64	1487.939	1487.939	1487.939	1487.939
2005	9	81	1487.939	1487.939	1487.939	1487.939
2006	10	100	1487.939	1487.939	1487.939	1487.939
2007	11	121	1487.939	1487.939	1487.939	1487.939
2008	12	144	1487.939	1487.939	1487.939	1487.939
2009	13	169	1487.939	1487.939	1487.939	1487.939
2010	14	196	1487.939	1487.939	1487.939	1487.939
2011	15	225	1487.939	1487.939	1487.939	1487.939
2012	16	256	1487.939	1487.939	1487.939	1487.939
2013	17	289	1487.939	1487.939	1487.939	1487.939
2014	18	324	1487.939	1487.939	1487.939	1487.939
2015	19	361	1487.939	1487.939	1487.939	1487.939
2016	20	400	1487.939	1487.939	1487.939	1487.939
2017	21	441	1487.939	1487.939	1487.939	1487.939

Table S7. Input dataset for Phosphorus Counterfactual A. Columns represent significant variables for the P regression model, Year, $Year^2$, $Year^3$, and the 4-year time-lagged P balance. Counterfactual A held the 1 balance term constant at 1975 levels after the appropriate lag of 4 years.

YEAR	YEAR_CENTER	YEAR2	YEAR3	P_BALANCE_KGSQKM_4LAGYR
1975	-21	441	-9261	424.5073
1976	-20	400	-8000	434.3323
1977	-19	361	-6859	454.3472
1978	-18	324	-5832	463.3422
1979	-17	289	-4913	406.3638
1980	-16	256	-4096	406.3638
1981	-15	225	-3375	406.3638
1982	-14	196	-2744	406.3638
1983	-13	169	-2197	406.3638
1984	-12	144	-1728	406.3638
1985	-11	121	-1331	406.3638
1986	-10	100	-1000	406.3638
1987	-9	81	-729	406.3638
1988	-8	64	-512	406.3638
1989	-7	49	-343	406.3638
1990	-6	36	-216	406.3638
1991	-5	25	-125	406.3638
1992	-4	16	-64	406.3638
1993	-3	9	-27	406.3638
1994	-2	4	-8	406.3638
1995	-1	1	-1	406.3638
1996	0	0	0	406.3638
1997	1	1	1	406.3638
1998	2	4	8	406.3638
1999	3	9	27	406.3638
2000	4	16	64	406.3638
2001	5	25	125	406.3638
2002	6	36	216	406.3638
2003	7	49	343	406.3638
2004	8	64	512	406.3638
2005	9	81	729	406.3638
2006	10	100	1000	406.3638
2007	11	121	1331	406.3638
2008	12	144	1728	406.3638
2009	13	169	2197	406.3638
2010	14	196	2744	406.3638
2011	15	225	3375	406.3638
2012	16	256	4096	406.3638
2013	17	289	4913	406.3638
2014	18	324	5832	406.3638
2015	19	361	6859	406.3638
2016	20	400	8000	406.3638
2017	21	441	9261	406.3638

Table S8. Input Dataset for Nitrogen Counterfactual B. Columns represent significant variables for the N regression model, Year, $Year^2$, and the 2, 4,9 and 11- year time-lagged nitrogen balances. Counterfactual B held the year terms constant from 1975 to 2017.

YEAR	YEAR_CENTER	YEAR2	N_BALANCE_KGSQKM_2LAGYR	N_BALANCE_KGSQKM_4LAGYR	N_BALANCE_KGSQKM_9LAGYR	N_BALANCE_KGSQKM_11LAGYR
1975	-21	441	1405.461	1416.574	945.8797	780.4774
1976	-21	441	1642.159	1384.349	1024.148	809.2727
1977	-21	441	1487.939	1405.461	1165.054	945.8797
1978	-21	441	1765.886	1642.159	1150.443	1024.148
1979	-21	441	1690.011	1487.939	1273.053	1165.054
1980	-21	441	1521.432	1765.886	1416.574	1150.443
1981	-21	441	1648.108	1690.011	1384.349	1273.053
1982	-21	441	1800.415	1521.432	1405.461	1416.574
1983	-21	441	1824.963	1648.108	1642.159	1384.349
1984	-21	441	1676.628	1800.415	1487.939	1405.461
1985	-21	441	1286.198	1824.963	1765.886	1642.159
1986	-21	441	1710.253	1676.628	1690.011	1487.939
1987	-21	441	1804.516	1286.198	1521.432	1765.886
1988	-21	441	1710.733	1710.253	1648.108	1690.011
1989	-21	441	1611.053	1804.516	1800.415	1521.432
1990	-21	441	1642.978	1710.733	1824.963	1648.108
1991	-21	441	1605.911	1611.053	1676.628	1800.415
1992	-21	441	1679.817	1642.978	1286.198	1824.963
1993	-21	441	1710.513	1605.911	1710.253	1676.628
1994	-21	441	1678.73	1679.817	1804.516	1286.198
1995	-21	441	1598.328	1710.513	1710.733	1710.253
1996	-21	441	1869.588	1678.73	1611.053	1804.516
1997	-21	441	1660.229	1598.328	1642.978	1710.733
1998	-21	441	1834.099	1869.588	1605.911	1611.053
1999	-21	441	1850.206	1660.229	1679.817	1642.978
2000	-21	441	1925.919	1834.099	1710.513	1605.911
2001	-21	441	1979.051	1850.206	1678.73	1679.817
2002	-21	441	1951.376	1925.919	1598.328	1710.513
2003	-21	441	1814.614	1979.051	1869.588	1678.73
2004	-21	441	1869.464	1951.376	1660.229	1598.328
2005	-21	441	1799.592	1814.614	1834.099	1869.588
2006	-21	441	1869.507	1869.464	1850.206	1660.229
2007	-21	441	1658.833	1799.592	1925.919	1834.099
2008	-21	441	1509.781	1869.507	1979.051	1850.206
2009	-21	441	1707.156	1658.833	1951.376	1925.919
2010	-21	441	1616.724	1509.781	1814.614	1979.051
2011	-21	441	1489.93	1707.156	1869.464	1951.376
2012	-21	441	1643.149	1616.724	1799.592	1814.614
2013	-21	441	1913.761	1489.93	1869.507	1869.464
2014	-21	441	2171.951	1643.149	1658.833	1799.592
2015	-21	441	2104.971	1913.761	1509.781	1869.507
2016	-21	441	1995.862	2171.951	1707.156	1658.833
2017	-21	441	1896.493	2104.971	1616.724	1509.781

Table S9. Input Dataset for Phosphorus Counterfactual B. Columns represent significant variables for the P regression model, Year, $Year^2$, $Year^3$, and the 4-year time-lagged P balance. Counterfactual B held the year terms constant from 1975 to 2017.

YEAR	YEAR_CENTER	YEAR2	YEAR3	P_BALANCE_KGSQKM_4LAGYR
1975	-21	441	-9261	424.5073
1976	-21	441	-9261	434.3323
1977	-21	441	-9261	454.3472
1978	-21	441	-9261	463.3422
1979	-21	441	-9261	406.3638
1980	-21	441	-9261	450.7354
1981	-21	441	-9261	458.097
1982	-21	441	-9261	392.2172
1983	-21	441	-9261	441.7751
1984	-21	441	-9261	429.6402
1985	-21	441	-9261	435.378
1986	-21	441	-9261	389.9033
1987	-21	441	-9261	326.6716
1988	-21	441	-9261	389.7277
1989	-21	441	-9261	364.3658
1990	-21	441	-9261	334.292
1991	-21	441	-9261	309.2501
1992	-21	441	-9261	318.4926
1993	-21	441	-9261	309.5167
1994	-21	441	-9261	323.9994
1995	-21	441	-9261	308.6334
1996	-21	441	-9261	307.0962
1997	-21	441	-9261	325.4516
1998	-21	441	-9261	326.8165
1999	-21	441	-9261	319.7014
2000	-21	441	-9261	336.606
2001	-21	441	-9261	355.4012
2002	-21	441	-9261	361.1587
2003	-21	441	-9261	324.8562
2004	-21	441	-9261	331.6136
2005	-21	441	-9261	330.6711
2006	-21	441	-9261	360.0186
2007	-21	441	-9261	319.1372
2008	-21	441	-9261	344.9561
2009	-21	441	-9261	314.2345
2010	-21	441	-9261	277.9631
2011	-21	441	-9261	284.0618
2012	-21	441	-9261	291.0029
2013	-21	441	-9261	221.843
2014	-21	441	-9261	325.5336
2015	-21	441	-9261	374.1542
2016	-21	441	-9261	409.4256
2017	-21	441	-9261	399.7543

Section 3 Results

Nutrient Balances

Table S10. Mean N and P inputs, outputs, and balances for the MRO for the time period 1950 to 2017. NA indicates not applicable.

Source	kg N km^{-2}	kg P km^{-2}
Inputs		
Fertilizer (Farm)	1604	308
Manure	1054	317
N-fixation	1287	NA
N-deposition	458	NA
Waste Water Treatment Facility Effluent	57	6
Fertilizer (Non-Farm)	23	5
Weathering	NA	7
Total Inputs	*4482*	*644*
Outputs		
Crop Uptake and Harvest Removal	2394	313
Gaseous Emissions	727	NA
Total Outputs	*3121*	*313*
(Inputs – Outputs) = Balance	1362	331

Trend Attribution

Regression Model

Diagnostic plots for the regression models developed to predict river TN and TP loads between 1975-2017 are presented here. The Bayesian Information Criterion (BIC) was used to select the model; the lowest BIC value is preferred (Schwarz 1978).

Figure S12. Regression model selection output criteria for A) Nitrogen and B) Phosphorus. Bayesian Information Criteria scores are shown on the y-axis and independent variables are shown on the x-axis. Each horizontal row corresponds to one model. Variables that are not included in a model are represented by white blocks. The model with the lowest BIC score is preferred, and black boxes indicate lower Y-axis values. The best N model includes 2, 4, 9, and 11-year time lagged N balances, Year^2, and Year terms. The best P model included 4-year time-lagged P balances, Year, Year^2, and Year^3 terms.

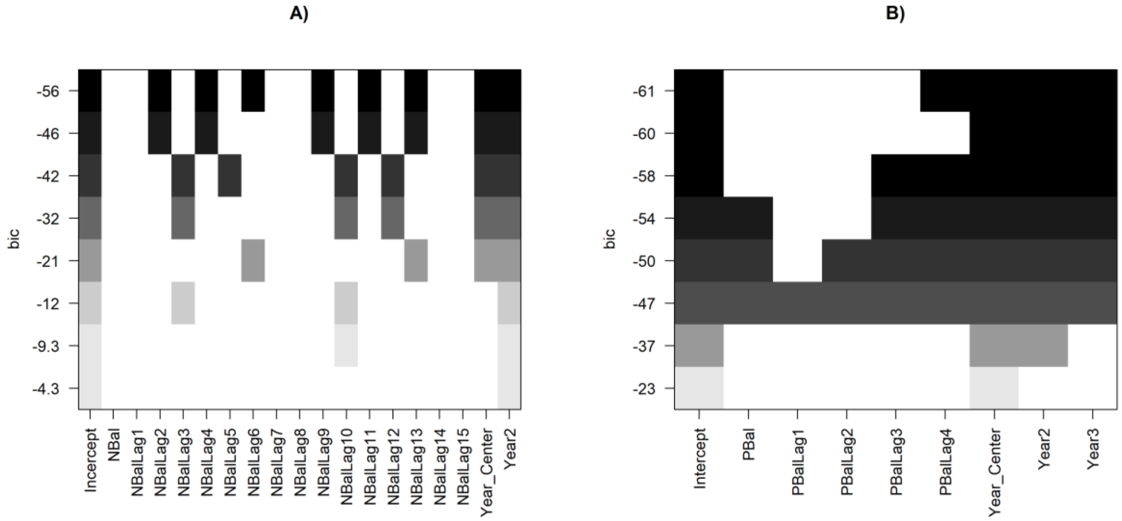

Figure S13. Regression model residual plots for predicting river A) TN loads, and B) TP loads at the MRO, 1975-2017.

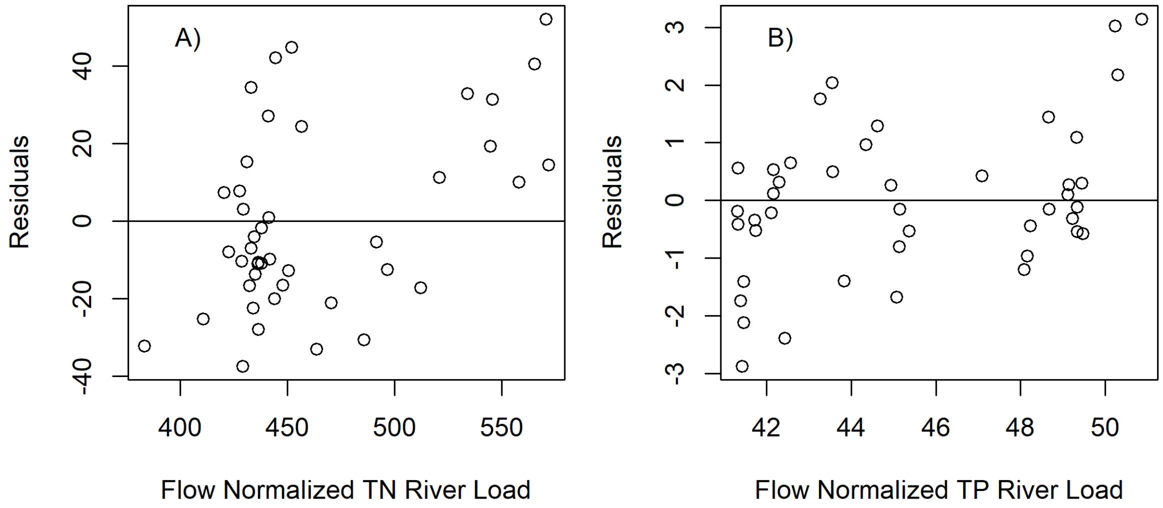

Figure S14. Observed river loads from WRTDS compared to predicted river loads from the regression model for A) nitrogen and B) phosphorus at the MRO, 1975-2017.

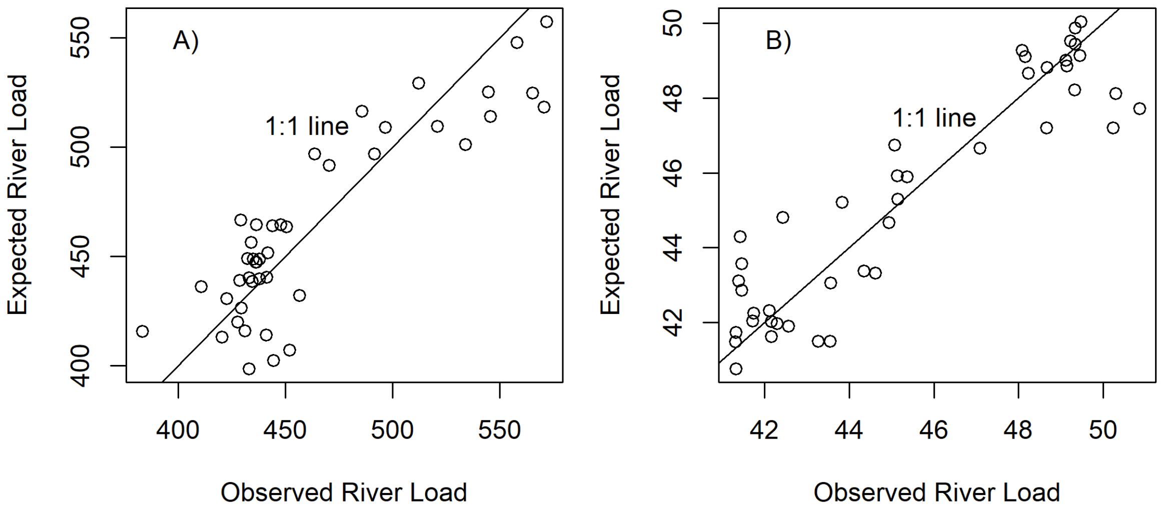

Figure S15. Comparison of annual WRTDS to river loads and 90% confidence intervals predicted from the regression model for A) Total Nitrogen, and B) Total Phosphorus. Model details for regression predicted river loads are specified in Table 3.

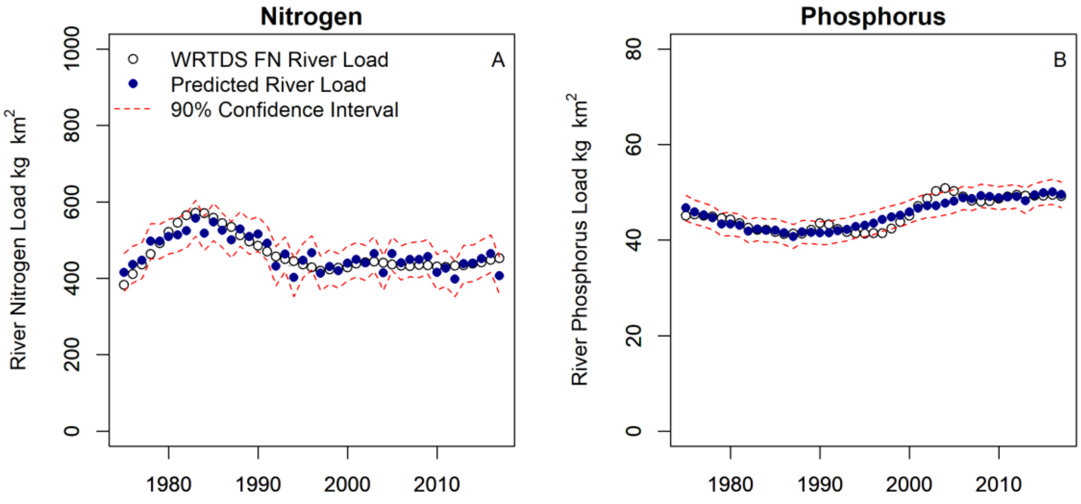

Section 4 Supporting data on tillage practices in the Mississippi River Basin

Table S11. Cultivation practice data in the MRB 1986 to 2017. The Percent Cropland in the MRB in the Conservation Reserve Program (Falcone 2017), and the percent of Cropland in the MRB under No Till and Intensive Tillage (Baker 2011).

Year	Percent Cropland in Conservation Reserve Program	Percent Cropland in No Till	Percent Cropland in Intensive Till
1986	0. 57	NA	NA
1987	3.5	NA	NA
1988	5.5	NA	NA
1989	6.7	6.7	45.2
1990	7.6	8.1	43.6
1991	7.7	8.8	42.2
1992	7.9	10.7	40.1
1993	8.1	13.5	35.2
1994	8.1	14.4	35.3
1995	8.1	15.5	35.1
1996	8.0	13.4	32.9
1997	7.6	17.2	32.2
1998	7.0	16.8	29.9
1999	6.8	NA	NA
2000	7.1	18.6	38.2
2001	7.5	NA	NA
2002	7.6	21.3	38.0
2003	7.7	NA	NA
2004	7.8	24.8	36.5
2005	8.3	NA	NA
2006	8.6	NA	NA
2007	8.7	NA	NA
2008	8.1	NA	NA
2009	7.9	NA	NA
2010	7.7	NA	NA
2011	7.6	NA	NA
2012	7.1	NA	NA
2013	6.4	NA	NA
2014	6.1	NA	NA
2015	6.1	NA	NA
2016	6.0	NA	NA
2017	5.8	45.9	25.6

Figure S16. Percentage of MRB A) Conservation Reserve Program (CRP), Tillage practices (No Till and Intensive Till), and Harvested Cropland, and B) just CRP.

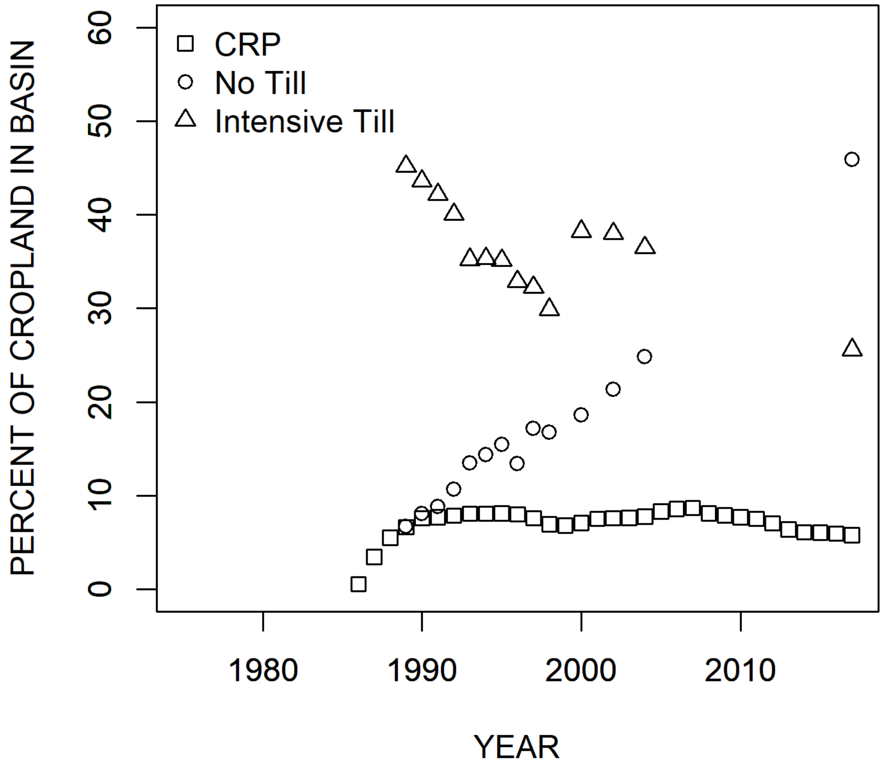

Section 5 - References

Alexander, R. B., and R. A. Smith. 1990. County-level estimates of nitrogen and phosphorus fertilizer use in the United States, 1945 to 1985. Citeseer.

Aulenbach, B. T., H. T. Buxton, W. T. Battaglin, and C. R.H. 2007. Streamflow and Nutrient Fluxes of the Mississippi-Atchafalaya River Basin and Subbasins for the Period of Record Through 2005, Sources and Preparation of Data Used for Nutrient Flux Estimation, U.S. Geological Survey Open-File Report 2007-1080, https://toxics.usgs.gov/pubs/of-2007-1080/sources.html.

Baker, N. T. 2011. Tillage practices in the conterminous United States, 1989-2004-Datasets Aggregated by Watershed. Report 573, Reston, VA.

Boyer, E. W., C. L. Goodale, N. A. Jaworski, and R. W. Howarth. 2002. Anthropogenic nitrogen sources and relationships to riverine nitrogen export in the northeastern USA. Biogeochemistry **57**:137-169.

Brakebill, J. W., and J. M. Gronberg. 2017 County-Level Estimates of Nitrogen and Phosphorus from Commercial Fertilizer for the Conterminous United States, 1987-2012: U.S. Geological Survey data release, https://doi.org/10.5066/F7H41PKX.

Coupe, R. H., D. A. Goolsby, W. A. Battaglin, J. K. Böhlke, P. B. McMahon, and C. Kendall. 2013. Transport of nitrate in the Mississippi River in July-August 1999. Annals of Environmental Science **7**.

Crawley, M. J. 2012. The R book. John Wiley & Sons.

De Klein, C., R. S. Novoa, S. Ogle, K. A. Smith, P. Rochette, T. C. Wirth, B. G. McConkey, A. Mosier, K. Rypdal, and M. Walsh. 2006. N2O emissions from managed soils, and CO2 emissions from lime and urea application. IPCC guidelines for National greenhouse gas inventories, prepared by the National greenhouse gas inventories programme **4**:1-54.

Falcone, J. A. 2017. Watershed characteristics for study sites of the Surface Water Trends project, National Water Quality Program: U.S. Geological Survey data release, https://doi.org/10.5066/F7TX3CKP.

Falcone, J. A. 2020. Tabular data for selected items from the Census of Agriculture for the period 1950-2017 for counties in the conterminous United States: U.S. Geological Survey data release, https://doi.org/10.5066/P9S4WQHU.

Falcone, J. A. 2021a. Estimates of county-level nitrogen and phosphorus from fertilizer and manure for approximately five-year periods from 1950 to 2017 for the conterminous United States: U.S. Geological Survey Open File Report, USGS Open File Report 2020-1153, 20 p., https://doi.org/10.3133/ofr20201153.

Falcone, J. A. 2021b. Tabular county-level nitrogen and phosphorus estimates from fertilizer and manure for approximately five-year periods from 1950 to 2017: U.S. Geological Survey data release, https://doi.org/10.5066/P9VSQN3C. .

Ferraro, P. J. 2009. Counterfactual thinking and impact evaluation in environmental policy. New directions for evaluation **2009**:75-84.

Gronberg, J. A. M., and T. Arnold. 2017. County-level estimates of nitrogen and phosphorus from animal manure for the conterminous United States, 2007 and 2012, USGS Open File Report 2017-1021.

Gronberg, J. M., and N. E. Spahr. 2012. County-level estimates of nitrogen and phosphorus from commercial fertilizer for the conterminous United States, 1987-2006. US Department of the Interior, US Geological Survey Reston, VA.

Harrell, F. E. 2019. Package 'hmisc', version 4.4-2. https://cran.r-project.org/web/packages/Hmisc/Hmisc.pdf. CRAN2018 **2019**:235-236.

Hartmann, J., N. Moosdorf, R. Lauerwald, M. Hinderer, and A. J. West. 2014. Global chemical weathering and associated P-release — The role of lithology, temperature and soil properties. Chemical Geology **363**:145-163.

Hirsch, R. M., S. A. Archfield, and L. A. De Cicco. 2015. A bootstrap method for estimating uncertainty of water quality trends. Environmental Modelling & Software **73**:148-166.
Hirsch, R. M., D. L. Moyer, and S. A. Archfield. 2010. Weighted regressions on time, discharge, and season (WRTDS), with an application to Chesapeake Bay river inputs 1. JAWRA Journal of the American Water Resources Association **46**:857-880.
Husson, F., and J. Josse. 2015. missMDA: Handling missing values with multivariate data analysis. R package version 1.9. https://CRAN.R-project.org/package=missMDA (accessed 25 Mar. 2016).
International Plant Nutrition Institute. 2012. A Nutrient Use Information System (NuGIS) for the U.S. Norcross, GA. January 12, 2012. www.ipni.net/nugis. Accessed, May 26, 2020.
Ivahnenko, T. I. 2017. Evaluation and use of US Environmental Protection Agency Clean Watersheds Needs Survey data to quantify nutrient loads to surface water, 1978–2012. 2328-0328, US Geological Survey.
Jordan, T. E., and D. E. Weller. 1996. Human contributions to terrestrial nitrogen flux. BioScience **46**:655-664.
Lamarque, J.-F., T. C. Bond, V. Eyring, C. Granier, A. Heil, Z. Klimont, D. Lee, C. Liousse, A. Mieville, B. Owen, M. G. Schultz, D. Shindell, S. J. Smith, E. Stehfest, J. Van Aardenne, O. R. Cooper, M. Kainuma, N. Mahowald, J. R. Mcconnell, V. Naik, K. Riahi, and D. P. Van Vuuren. 2010. Historical (1850–2000) gridded anthropogenic and biomass burning emissions of reactive gases and aerosols: methodology and application. Atmospheric Chemistry and Physics **10**:7017-7039.
LaMotte, A. E. 2015 Selected items from the Census of Agriculture at the county level for the conterminous United States, 1950-2012: U.S. Geological Survey data release, http://dx.doi.org/10.5066/F7H13016.
Lee, C. J., J. C. Murphy, C. G. Crawford, and J. R. Deacon. 2017. Methods for computing water-quality loads at sites in the U.S. Geological Survey National Water Quality Network. Report 2017-1120, Reston, VA.
Lumley, T. 2020. Package "leaps" version 3.1. , https://cran.r-project.org/web/packages/leaps/leaps.pdf, Accessed August 1, 2020. .
McIsaac, G. F., M. B. David, G. Z. Gertner, and D. A. Goolsby. 2002. Relating net nitrogen input in the Mississippi River Basin to nitrate flux in the Lower Mississippi River. Journal of Environmental Quality **31**:1610-1622.
Mueller, D. K., and J. M. Gronberg. 2013. County-level estimates of nitrogen and phosphorus from animal manure for the conterminous United States, 2002: U.S. Geological Survey Open-File Report 2013–1065, https://pubs.usgs.gov/of/2013/1065/.
Murphy, J., R. M. Hirsch, and L. A. Sprague. 2013. Nitrate in the Mississippi River and its tributaries, 1980-2010: An update. US Geological Survey.
Murphy, J., and L. Sprague. 2019. Water-quality trends in US rivers: Exploring effects from streamflow trends and changes in watershed management. Science of the Total Environment **656**:645-658.
Oelsner, G. P., L. A. Sprague, J. C. Murphy, R. E. Zuellig, H. M. Johnson, K. R. Ryberg, J. A. Falcone, E. G. Stets, A. V. Vecchia, M. L. Riskin, L. A. De Cicco, T. J. Mills, and W. H. Farmer. 2017. Water-quality trends in the Nation's rivers and streams, 1972–2012—Data preparation, statistical methods, and trend results (ver. 2.0, October 2017): U.S. Geological Survey Scientific Investigations Report 2017–5006, 136 p., https://doi.org/10.3133/sir20175006.
Robertson, D. M., and D. A. Saad. 2019. Spatially referenced models of streamflow and nitrogen, phosphorus, and suspended-sediment loads in streams of the midwestern United States. Report 2019-5114, Reston, VA.
Robertson, G. P., and P. Groffman. 2007. Nitrogen transformations. Pages 341-364 Soil microbiology, ecology and biochemistry. Elsevier.
Russell, M. J., D. E. Weller, T. E. Jordan, K. J. Sigwart, and K. J. Sullivan. 2008. Net anthropogenic phosphorus inputs: spatial and temporal variability in the Chesapeake Bay region. Biogeochemistry **88**:285-304.

Sabo, R. D., C. M. Clark, J. Bash, D. Sobota, E. Cooter, J. P. Dobrowolski, B. Z. Houlton, A. Rea, D. Schwede, S. L. Morford, and J. E. Compton. 2019. Decadal Shift in Nitrogen Inputs and Fluxes Across the Contiguous United States: 2002–2012. Journal of Geophysical Research: Biogeosciences **124**:3104-3124.

Schwarz, G. 1978. Estimating the dimension of a model. Annals of statistics **6**:461-464.

Smith, D. B. C., W. F. Woodruff, L. G. Solano, F. Ellefsen, and J. Karl. 2014. Geochemical and mineralogical maps for soils of the conterminous United States.

Sprague, L. A., and J. A. M. Gronberg. 2012. Relating Management Practices and Nutrient Export in Agricultural Watersheds of the United States. Journal of Environmental Quality **41**:1939-1950.

Sprague, L. A., R. M. Hirsch, and B. T. Aulenbach. 2011. Nitrate in the Mississippi River and Its Tributaries, 1980 to 2008: Are We Making Progress? Environmental Science & Technology **45**:7209-7216.

Swaney, D. P., R. W. Howarth, and B. Hong. 2018. Nitrogen use efficiency and crop production: Patterns of regional variation in the United States, 1987–2012. Science of the Total Environment **635**:498-511.

Terziotti, S. 2019. Distribution of phosphorus in soils and aggregated within geologic mapping units, conterminous United States: U.S. Geological Survey data release, https://doi.org/10.5066/P918DF1E.

Turner, R. E., N. N. Rabalais, and D. Justic. 2006. Predicting summer hypoxia in the northern Gulf of Mexico: Riverine N, P, and Si loading. Marine Pollution Bulletin **52**:139-148.

US Environmental Protection Agency. 2020. Critical Loads Mapper Tool, https://www.epa.gov/air-research/critical-loads-mapper-tool (Accessed, May 7, 2020).

US Geological Survey. 2019. Mississippi River near St. Francisville, LA, Technical Information Page, http://kswsc.cr.usgs.gov/~cjlee/mississippi_loads_trend2020all/#/TECH (accessed October 18, 2019).

Withers, P. J. A., and H. P. Jarvie. 2008. Delivery and cycling of phosphorus in rivers: A review. Science of the Total Environment **400**:379-395.
